# Two novel cyanobacterial α-dioxygenases for the biosynthesis of fatty aldehydes

**DOI:** 10.1007/s00253-021-11724-x

**Published:** 2021-12-09

**Authors:** In Jung Kim, Yannik Brack, Thomas Bayer, Uwe T. Bornscheuer

**Affiliations:** Department of Biotechnology and Enzyme Catalysis, Institute of Biochemistry, University of Greifswald, 17489 Greifswald, Germany

**Keywords:** α-Dioxygenase, Fatty aldehyde, Fatty acid, *Cyanobacteria*, Aroma compounds

## Abstract

α-Dioxygenases (α-DOXs) are known as plant enzymes involved in the α-oxidation of fatty acids through which fatty aldehydes, with a high commercial value as flavor and fragrance compounds, are synthesized as products. Currently, little is known about α-DOXs from non-plant organisms. The phylogenic analysis reported here identified a substantial number of α-DOX enzymes across various taxa. Here, we report the functional characterization and *Escherichia coli* whole-cell application of two novel α-DOXs identified from cyanobacteria: CalDOX from *Calothrix parietina* and LepDOX from *Leptolyngbya* sp. The catalytic behavior of the recombinantly expressed CalDOX and LepDOX revealed that they are heme-dependent like plant α-DOXs but exhibit activities toward medium carbon fatty acids ranging from C10 to C14 unlike plant α-DOXs. The in-depth molecular investigation of cyanobacterial α-DOXs and their application in an *E. coli* whole system employed in this study is useful not only for the understanding of the molecular function of α-DOXs, but also for their industrial utilization in fatty aldehyde biosynthesis.

**Key points**

• *Two novel α-dioxygenases from Cyanobacteria are reported*

• *Both enzymes prefer medium-chain fatty acids*

• *Both enzymes are useful for fatty aldehyde biosynthesis*

**Graphical abstract:**

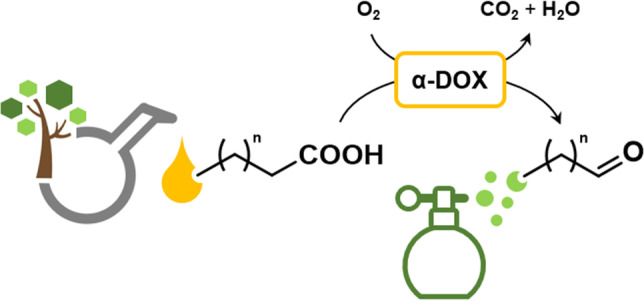

**Supplementary Information:**

The online version contains supplementary material available at 10.1007/s00253-021-11724-x.

## Introduction

Fatty aldehydes represent a structurally diverse group of aliphatic compounds derived from the corresponding fatty acids. They occur in a wide range of living organisms including plants, insects, and mammals as metabolic intermediates of lipids (Foster and Anderson 2019; Gilbertson et al. 1972; Liu et al. 2012). Many types of fatty aldehydes provide pleasant and diverse odor impressions depending on their chemical structure determined by chain length, the presence/number/position of double bonds, and further substitutions (Hammer et al. 2020). Thus, they are widely applied in the food, perfume, cosmetic, and detergent industries as important flavor and fragrance compounds.

Extraction from natural sources, such as plants and fungi, has been traditionally achieved to obtain fatty aldehydes, and the in-depth analysis of these extracts has served as a useful basis for the identification of valuable compounds (Hammer et al. 2021). Currently, most of commercial flavors and fragrances are chemically synthesized using either fossil-based starting materials or renewable lipid sources (Gaylord 1957). However, this often requires hazardous chemicals and energy-demanding high-temperature and high-pressure conditions. In contrast, biotechnological routes can be a more effective way to accomplish “greener” and practical production of fatty aldehydes. More importantly, they can meet the consumer’s preference for naturally produced products (Kunjapur and Prather 2015).

To date, most of the biosynthetic pathways for fatty aldehydes — either as the final product or as precursors to industrially relevant compounds — have been devised by introducing fatty acid acyl-CoA/ACP reductases (FARs) (Foo et al. 2020; Lehtinen et al. 2018, 2017; Reiser and Somerville 1997) or carboxylic acid reductases (CARs) (Akhtar et al. 2013; Maurer et al. 2019; Wu et al. 2021). Many attempts were made for the de novo synthesis of fatty aldehydes by introducing FAR in various organisms such as *Escherichia coli*, *Saccharomyces cerevisiae*, or *Acinetobacter baylyi* (Foo et al. 2020; Lehtinen et al. 2018, 2017; Reiser and Somerville 1997). For these approaches, however, activated fatty acid (AFA) precursors, typically CoA-derivatives, are required as substrate, and the FAR enzyme activity is often too low to generate industrially sufficient yields. The utilization of free fatty acids (FFAs) instead of AFAs as the substrate possesses advantages since FFAs are more abundant in biological systems and can be readily supplied from cheap renewable resources (Hu et al. 2020). The alternative strategy most known to complement the FAR-based pathway is based on CARs (Akhtar et al. 2013; Hu et al. 2020; Maurer et al. 2019; Wu et al. 2021). CARs are not only capable of directly acting on FFA but also are versatile enzymes with broad substrate spectra. However, issues related to technical complexity and expensive process cost cannot be avoided when using this enzyme class as they require the cofactors ATP, NADPH, and Mg^2+^ and the additional expression of a partner enzyme, a phosphopantetheinyl transferase (PPTase) (Akhtar et al. 2013; Maurer et al. 2019; Wu et al. 2021). In contrast, α-dioxygenases (α-DOXs), a family of heme-dependent fatty acid dioxygenases that convert FFAs into fatty aldehydes, can be potent candidates to tackle these problems as they require only molecular oxygen, but no expensive cofactors (Goulah et al. 2013; Hamberg et al. 2002; Hammer et al. 2020; Kaehne et al. 2011; Koeduka et al. 2005).

Biologically, α-DOXs catalyze the initial step of fatty acid α-oxidation in plants for the synthesis of oxylipin and, hence, are of physiological significance (Mosblech et al. 2009). The expression of α-DOX has been upregulated as a means of a defense mechanisms against various kinds of biotic and abiotic stress factors including pathogens, oxidative and cold stress, and osmosis, for example (Koeduka et al. 2005). In the α-DOX–mediated α-oxidation of a fatty acid (C_n_), molecular oxygen is accepted as the cosubstrate and inserted at an α-methylene carbon to produce (*R*)-2-hydroperoxy fatty acids (C_n_) as intermediate, which spontaneously decarboxylates, yielding the fatty aldehydes (C_n-1_) and also the (*R*)-2-hydroxy fatty acid (C_n_) (Hamberg et al. 2002; Mukherjee et al. 2011).

Although this fatty acid α-oxidation mechanism is well-known in plants and, accordingly, catalytic functions of various α-DOXs from plants have been thoroughly elucidated, there is only one functional study of an α-DOX from a non-plant organism, the CsDOX from the cyanobacterium *Crocosphaera subtropica* (Hammer et al. 2020). CsDOX was revealed to have a catalytically equivalent function to α-DOX from plants but shows a different substrate specificity. This report motivated us to explore the functional diversity of α-DOXs from non-plant organism and to evaluate their catalytic potentials in whole-cell systems. In this study, we identified two novel α-DOXs from the cyanobacteria *Calothrix parietina* (CalDOX) and *Leptolyngbya* sp. (LepDOX) via InterPro-based domain search (Hunter et al. 2009), and we demonstrate the in-depth molecular characterization of these enzymes. Furthermore, using an *E. coli* whole-cell system, the CalDOX- or LepDOX-mediated microbial synthesis of fatty aldehydes from readily available cheap substrates was achieved. The results obtained from this study will provide not only new insights into the molecular function of non-plant α-DOXs but also enable the biotechnological production of fatty aldehydes and/or fatty aldehyde–derived biochemicals using these enzymes.

## Materials and methods

### Phylogenetic tree analysis

An InterPro-based search using AtDOX1 from *Arabidopsis thaliana* as the query (GenBank accession no. AAK68727.1) identified approximately 1400 known or putative α-DOX family members from genome-sequenced organisms (Apweiler et al. 2001). Sequences with > 1000 amino acids in length as well as redundant (CD-HIT, a cutoff with 0.99) (Huang et al. 2010) and obviously faulty sequences were discarded. Among the sequences originating from various organisms, one from each genus was selected for the construction of a phylogenetic tree. The resulting amino acid sequences were aligned using MUSCLE (Edgar 2004), after which the alignment was trimmed by removing N′- (0–368) and C′- (980–1288) extensions. After two rounds of alignment optimization, the final alignment was obtained. The best model was determined through ModelFinder (WAG + R8) (Kalyaanamoorthy et al. 2017), subsequently applied for tree building using IQTree (http://www.iqtree.org/), and finally visualized by FigTree (http://tree.bio.ed.ac.uk/software/figtree/).

### Expression and purification

Codon-optimized genes of CalDOX (GenBank accession no. MZ522724) and LepDOX (GenBank accession no. MZ522725) were synthesized (BioCat, Heidelberg, Germany) (Supplemental Fig. S1) and were cloned into the expression vector pET28a (Novagen, Darmstadt, Germany). The recombinant genes for putative α-DOXs were transformed into competent *E. coli* BL21 (DE3) cells. *E. coli* cells containing α-DOX genes were cultivated in LB (Lysogeny Broth) medium containing 50 μg/mL kanamycin. The induction of protein expression was performed by adding 0.5-mM isopropyl β-D-1-thiogalactopyranoside (IPTG). Cells were further incubated at 18 °C for 18 h and were harvested by centrifugation at 4500 × *g* and 4 °C for 20 min. Then, cell pellets were resuspended in buffer A (50-mM Tris–HCl, 200-mM NaCl, and 20-mM imidazole; pH 8.0) and disrupted by an ultrasonicator. Only during cell lysis for α-DOX, 1% (v/v) of Triton X-100 was supplemented to facilitate extraction. The whole-cell lysate was centrifuged twice at 10,000 × *g* and 4 °C for 30 min. The supernatant containing soluble crude extract was loaded onto a column containing Ni-agarose resin (Carl Roth, Karlsruhe, Germany) and purified by immobilized metal ion affinity chromatography (IMAC). The His-tagged proteins were eluted by the addition of buffer B (50-mM Tris–HCl, 200-mM NaCl, and 300-mM imidazole; pH 8.0). The proteins were analyzed to check expression and purity by sodium dodecyl sulfate–polyacrylamide gel electrophoresis (SDS-PAGE). The protein concentrations were quantified by Bradford assay using bovine serum albumin (BSA) as the standard (Carl Roth). For further purification and the analysis of oligomeric state of CalDOX and LepDOX, size-exclusion chromatography (SEC) was performed with a HiPrep 16/60 Sephacryl S-200 HR column (Cytiva, Marlborough, MA, USA) using a buffer containing 10-mM Tris–HCl and 200-mM NaCl (pH 8.0) as the mobile phase.

### Oxygen depletion assay

Oxygenase activities of α-DOX enzymes were measured by an oxygen consumption assay. Unless otherwise mentioned, the enzyme reaction was conducted as follows using the fluorescence-based 96-well oxygen sensor, OxoPlate OP96U (PreSens, Regensburg, Germany): The enzyme reaction containing 2-mM myristic acid in 0.1-M Tris–HCl (pH 7.0) with 1% (v/v) Triton X-100 in a total volume of 270 μL was initiated by the addition of 3 μL purified CalDOX or LepDOX (0.07 to 1 μg). After sealing with transparent adhesive foil, the oxygen sensor plate was immediately put into a Varioskan™ LUX multimode plate reader (Thermo Fisher Scientific, Darmstadt, Germany). According to the manufacturer’s instructions, oxygen consumption was monitored by fluorescence measurement at 25 °C with dual kinetic and bottom reading modes using two different filter pairs for the fluorescence detection from indicator and reference dyes. For both dyes, 540 nm was used for excitation and fluorescence emission measured at 650 and 590 nm for indicator and reference dyes, respectively. A two-point calibration was constructed using air-saturated and air-free water standards. Based on the equation provided by the manufacturer, the dissolved oxygen level in the reaction mixture was calculated, which was then subtracted from the saturated oxygen level to obtain the amount of oxygen consumed by enzyme reactions. One unit was defined as the amount of enzyme that consumes 1 μmole of oxygen per minute under the specified condition.

### Effects of temperature, pH, and metal ions

The effect of temperature on enzyme activity was investigated by incubating 3.3 μg of purified CalDOX with 2-mM myristic acid. Enzymatic reactions were performed under various temperature conditions ranging from 20 to 40 °C with an interval of 5 °C at pH 7 (0.1-M Tris–HCl) and 1000 rpm for 20 min in a total volume of 300 μL in the absence of Triton X-100. Then, 300 μL of hexane was added to the reaction mixture for the extraction of fatty aldehydes, followed by centrifugation at 10,000 × *g* for 1 min to separate the two phases. After taking the organic phase, enzyme activity was determined by quantification of tridecanal produced from the reaction via GC-FID analysis.

To determine the effects of pH and metal ions, the oxygen depletion assay was used. For the pH effect, enzyme reactions were carried out with 2-mM myristic acid in a buffer containing 1% (v/v) Triton X-100, initiated by purified CalDOX or LepDOX (0.1 to 1 μg) at 25 °C. The buffers used were 0.1-M sodium acetate (pH 4 to 6), 0.1-M sodium phosphate (pH 6 to 7), 0.1-M Tris–HCl (pH 7 to 9), and 0.1-M glycine–NaOH (pH 9 to 10). The effect of metal ions was investigated with 2-mM myristic acid in a buffer containing 1% (v/v) Triton X-100 by incubating with purified CalDOX or LepDOX (0.1 to 1 μg) at 25 °C and pH 7 (0.1-M Tris–HCl) in the presence of 1-mM magnesium chloride (MgCl_2_), zinc chloride (ZnCl_2_), copper chloride (CoCl_2_), cobalt chloride (CoCl_2_), ferric chloride (FeCl_3_), manganese sulfate (MnSO_4_), or calcium chloride (CaCl_2_). Activities were determined at the initial time phase and experiments were conducted in more than three independent replicates.

### Whole-cell biotransformation

Expression of recombinant CalDOX and LepDOX enzyme was induced in *E. coli* cells as described above. After harvesting cells by centrifugation at 9600 × *g* and 4 °C for 10 min, cells were washed with a resting cell medium (RCM) containing 200-mM potassium phosphate and 50-mM NaCl (pH 7.4), followed by centrifugation under the same conditions. After decanting the supernatant, cells were resuspended in the RCM.

Whole-cell biotransformations were conducted with *E. coli* resting cells at 12.5 g wet weight per L RCM at 35 °C and 1000 rpm with a reaction volume of 300 μL using a ThermoMixer (Eppendorf, Hamburg, Germany). The reaction was initiated by adding 15 μL of 100-mM fatty acid substrates (C10:0 or C14:0) from a DMSO stock solution to 285 μL of the resting cell suspension. After incubating for the indicated times, the biotransformations were stopped by the addition of 30-μL 2-M HCl. The samples were extracted with an equal volume of ethyl acetate three times and dried over anhydrous Na_2_SO_4_. The collected organic phases were subjected to GC-FID analysis.

### Gas chromatography analysis

Sample analysis was performed through a gas chromatography (GC) system (Shimadzu, Nakagyo-ku, Kyoto, Japan) with a flame ionization detector (FID) using a ZB‐5MSi capillary column (30 m × 0.25 mm, 0.25-µm film thickness; Phenomenex, Torrance, CA, USA) as the stationary phase and H_2_ as the carrier gas. The linear velocity of the mobile phase was set to 42.2 cm/s. A 1 µL sample was injected into the GC inlet in split mode with a ratio of 10, and the injection port temperature was set at 300 °C. The temperature program of the oven started from 80 °C for 5 min and then increased to 240 °C with a rate of 8 °C/min, and this was held for 8 min. For identification and quantification of fatty acids and fatty aldehydes, the corresponding standards at different concentrations were prepared, and calibration curves were constructed.

### Structural modeling and sequence alignment

The amino acid sequences of CalDOX and LepDOX were analyzed by alignment with two structurally and functionally known α-DOXs from *A. thaliana* (AthDOX1; GenBank accession no. AAK68727.1 and PDB code, 4KVK) and *Oryza sativa* (OsaDOX; GenBank accession no. ABA98060.2 and PDB code, 4HHS). Sequence alignment was performed using Clustal Omega (Sievers and Higgins 2014) and ESPript (Gouet et al. 1999). Homology model structures of CalDOX and LepDOX were constructed using the crystal structure of wild-type OsaDOX (PDB code: 4HHS) based on SWISS-MODEL (http://swissmodel.expasy.org/). The illustration of structures was made by PyMOL (http://www.pymol.org).

## Results

### Phylogenetic tree of α-DOX family

**Fig. 1:**
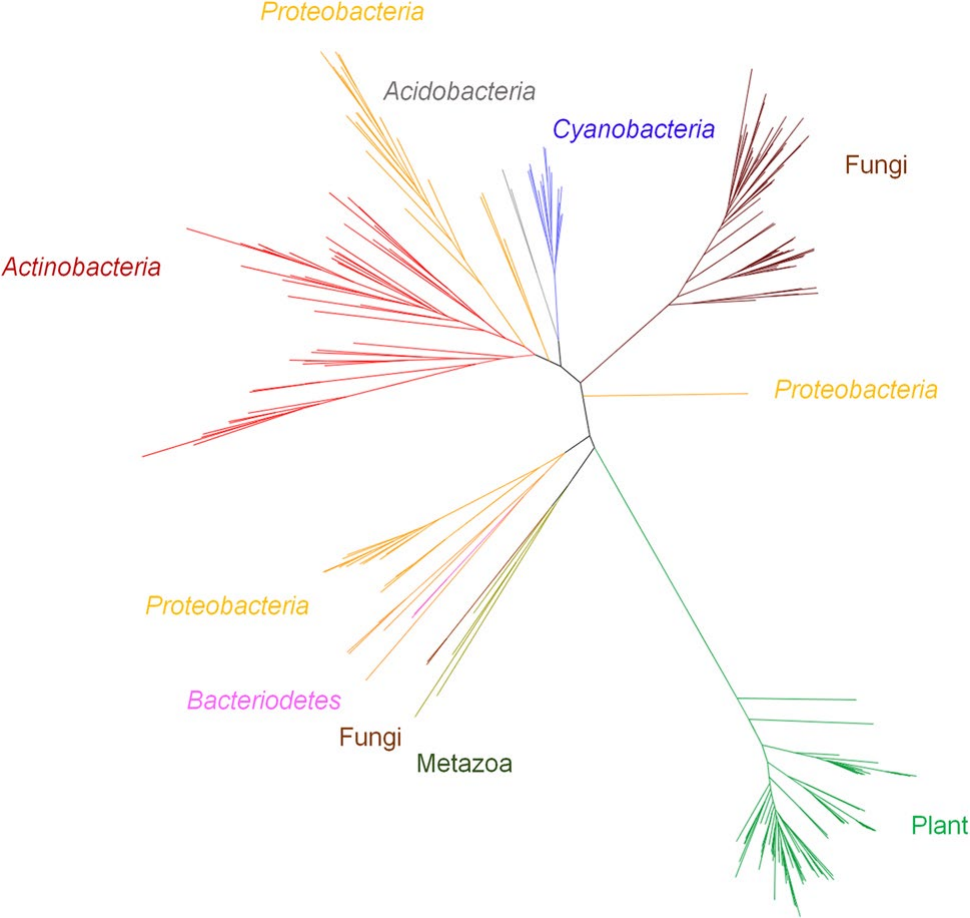
Radial phylogenetic tree of α-DOX family searched based on InterPro analysis. The α-DOX sequences were widely distributed among various organisms including bacteria (*Cyanobacteria*, *Proteobacteria*, *Actinobacteria*, *Acidobacteria*, and *Bacteroidetes*), fungi, plants, and metazoa

The phylogenetic analysis revealed that disparate clusters of α-DOX sequences distributed over a range of bacteria, fungi, and metazoa in addition to plants, none of which has so far been reported to include α-DOX (Fig. 1). In five bacterial phyla including *Cyanobacteria*, *Proteobacteria*, *Actinobacteria*, *Acidobacteria*, and *Bacteroidetes*, α-DOXs were found, in which α-DOXs from *Cyanobacteria* and *Actinobacteria* were clustered together, while those from *Proteobacteria* were dispersed throughout the tree. α-DOX–possessing fungi were restricted to the phylum *Ascomycota*. In case of plants, α-DOXs were found not only in higher plants but also in primitive land plants, such as moss (Bryophyta). The amino acid sequence analysis shows that all sequences contain the conserved catalytic tyrosine, which has an essential role in the oxygen insertion into the fatty acid. Regarding the two heme ligands playing a role in generating the radical of catalytic tyrosine, the proximal histidine residue was also conserved, whereas the distal histidine was partially conserved.

Among these newly identified α-DOX sequences, CalDOX and LepDOX from non-pathogenic cyanobacteria *Calothrix parietina* and *Leptolyngbya* sp., respectively, were chosen for biochemical investigation in this work.

### CalDOX and LepDOX are hemoproteins

**Fig. 2:**
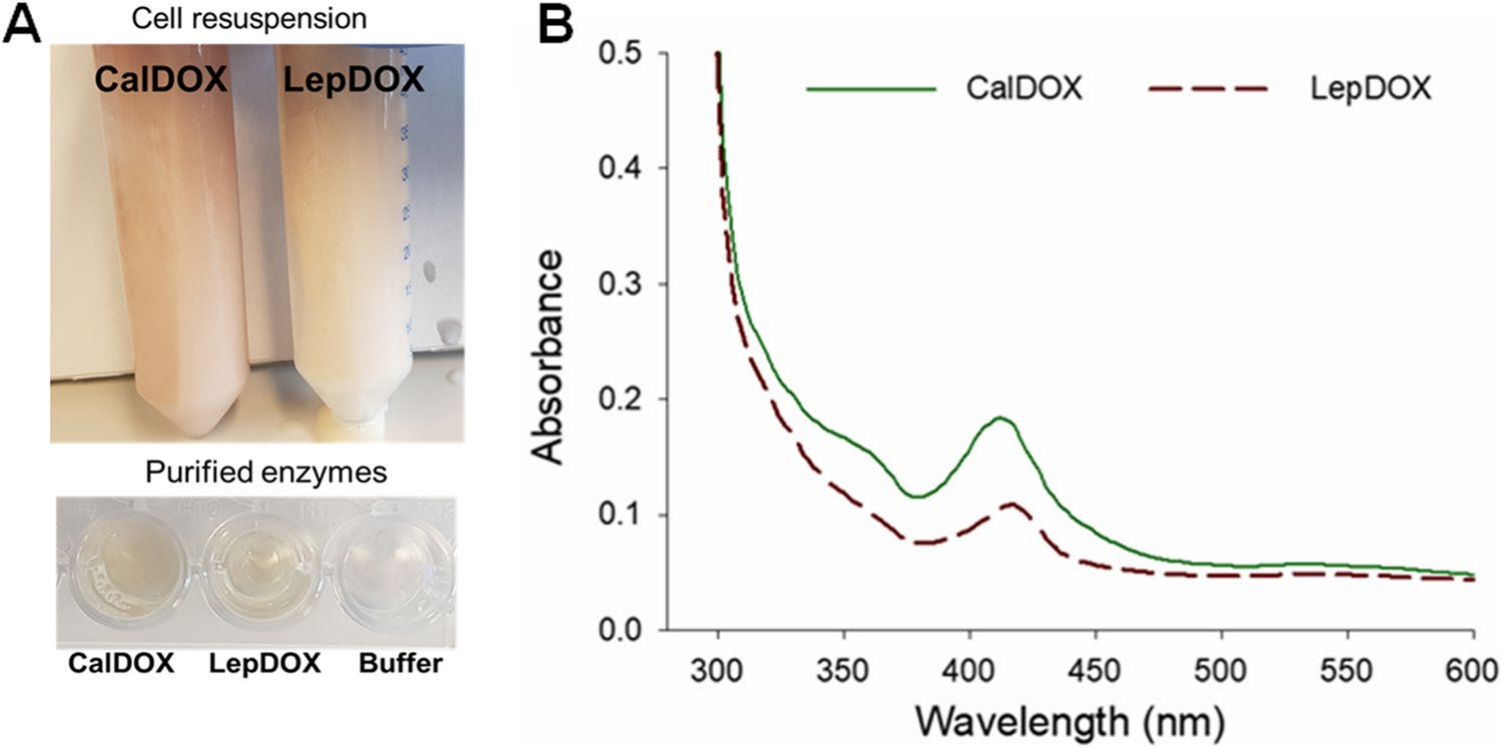
Identification of cyanobacterial α-DOXs as hemeproteins. **a** Cell resuspension for CalDOX and LepDOX and their purified forms show red colors. **b** Identification of the Soret peak with purified CalDOX and LepDOX by UV–Vis spectroscopy

Plant α-DOXs are known to be hemeproteins (Goulah et al. 2013; Liu et al. 2004; Mukherjee et al. 2011; Zhu et al. 2013), which utilize heme as the indispensable constituent to perform their oxygenase activity. In our study, the cell pellets of *E. coli* with recombinantly expressed α-DOX enzymes as well as their purified forms exhibited a red color, indicating that α-DOX derived from cyanobacteria also possesses a heme (Fig. 2). To further elucidate this, the absorbance spectra of the purified enzymes were scanned using UV–Vis spectroscopy. As a result, CalDOX and LepDOX showed Soret peaks at 412 nm, which is the typical property of plant α-DOXs (Fig. 2). From these results, the cyanobacterial α-DOXs newly identified in our study were found to be functionally equivalent to plant α-DOXs in terms of their heme-dependency, which was not elucidated for the previously described cyanobacterial CsDOX (Hammer et al. 2020).

Despite the obvious identification of CalDOX and LepDOX as heme proteins, high variation in Soret peak intensities were observed for every cultivation batch, with A_412_/A_280_ ratios ranging from 0.03 to 0.2, meaning a high batch-to-batch variation of heme incorporation into the enzymes. In every cultivation, samples had different proportion of heme-containing active proteins. In an effort to saturate heme incorporation, growth media were supplemented with 5-aminolevulinic acid (5-ALA), FeCl_3_, 5-ALA and FeCl_3_, or hemin during the synthesis of cyanobacterial α-DOXs. However, the supplementation did not improve the heme occupancy significantly (data not shown). Next, when hemin was added to purified CalDOX, a slightly higher intensity of the Soret peak was observed, indicating that heme can indeed be incorporated even into the already formed CalDOX protein. Still, the heme incorporation appears to be insufficient to achieve full saturation (Supplemental Fig. S2).

### Oligomeric states of CalDOX and LepDOX

**Fig. 3:**
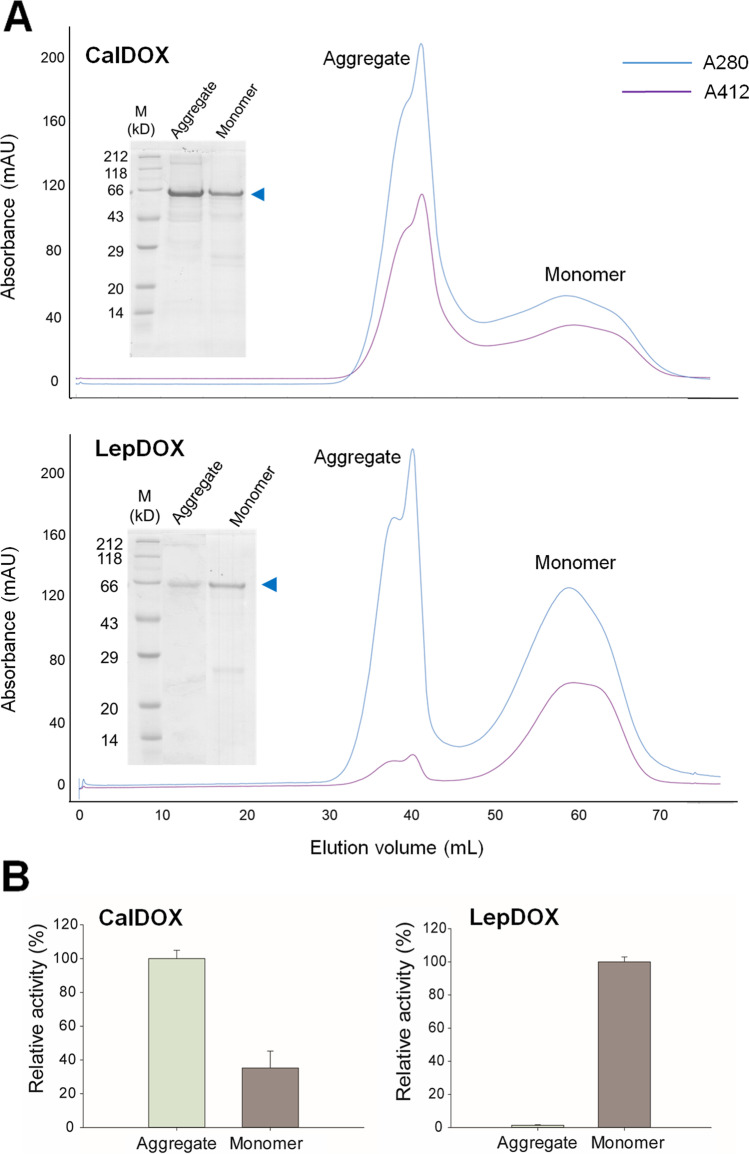
**a** Size-exclusion chromatography profiles for CalDOX and LepDOX. Insets show SDS-PAGE analyses of the aggregate and monomeric forms of CalDOX and LepDOX. M represents the protein marker. **b** Oxygen consumption activities of the aggregated and monomeric forms of CalDOX and LepDOX. Oxygen consumption activities were determined by incubating 2-mM myristic acid with the aggregated and monomeric forms of enzymes (0.07 to 1 μg) at 25 °C and pH 7

Although plant α-DOXs are functionally known as monomers (Goulah et al. 2013; Zhu et al. 2013), oligomeric states of AtDOX can highly vary with the expression and detergent systems from 1 to 10 subunits (Liu et al. 2006). Here, the absorbances at 280 and 412 nm were used for the monitoring of the total protein content and heme-bound protein, respectively, during the size-exclusion chromatographic run. Also, *R*_z_ values, the ratio of absorbance at the Soret peak to the absorbance at 280 nm (Colas and De Montellano 2004), for monomeric and aggregated fractions of CalDOX and LepDOX pooled from SEC were determined (Table S2). Two major peaks corresponding to the aggregated and monomer forms were revealed for both CalDOX and LepDOX (Fig. 3a). Each form had the same monomer molecular mass of around 67 kDa on SDS-PAGE. In both aggregate and monomer forms, heme-association with the proteins was identified with CalDOX with similar values of *R*_z_ (Fig. 3a and Table S2). In contrast, whereas heme-binding was clearly observed in the monomer form in LepDOX with similar *R*_z_ values to that of aggregates and monomers of CalDOX, the occupancy of the heme in the aggregated form of LepDOX was significantly lower, as evidenced by the 2.8-fold lower Soret peak intensity compared to that of the monomeric form at the same protein amount (gram-based), that is, a lower *R*_z_ value (Fig. 3a and Table S2). In case of CalDOX, both aggregate and monomer fractions showed oxygen-consuming activity with myristic acid, indicating that the aggregate maintains an intact catalytic machinery of CalDOX (Fig. 3b; left). However, LepDOX in aggregate form shows little activity, whereas its monomer shows oxygenase activity (Fig. 3b; right). This indicates that aggregate formation led to disruption of the catalytic machinery for LepDOX, possibly by affecting the enzyme’s heme environment. Thus, only the monomeric form of LepDOX was used for the enzyme assay. These results provide evidence that the presence or absence of heme in the aggregated form of cyanobacterial α-DOXs could be highly associated with their catalytic activity. However, the possibility that structural configurations of aggregates also can affect their catalytic machinery cannot be excluded.

To better understand the status of oligomeric states of the enzymes, native gel analyses were performed (Supplemental Fig. S3). Although it is difficult to estimate the exact molecular mass of oligomers on native gels, a rough information on the native states of the enzymes could be obtained. The aggregate fractions in both enzymes were found to be heterogeneous mixtures, which was not obviously from SEC analyses. For the monomer fractions, in contrast to the native CalDOX showing only one major band, LepDOX consisted of two major bands. The existence of the LepDOX monomer as two different species can be explained by the possibility that non-heme–bound and heme-bound species in the LepDOX monomer fraction may have different charges that lead to distinct migration rates according to native PAGE.

### Effect of Triton X-100

In this study, Triton X-100 has been used for cyanobacterial α-DOX enzymes during cell lysis. The extraction yields (mg protein/L cell culture) for CalDOX and LepDOX were improved approximately 2.5- and 1.1-fold, respectively when 1% (v/v) of Triton was added during cell disruption. Interestingly, both purified enzymes from the cells treated with Triton resulted in a stronger (~ 1.6-fold) and blue-shifted Soret peak at the same protein concentration (Table S1). With a higher and shifted Soret peak, the enzymes showed a 1.2-fold higher oxygenase activity (Table S1). This may be explained by the fact that the detergent can behave as a membrane-like environment, maintaining the conformation of membrane proteins and its heme environment similar to the native state (Noordermeer et al. 2001).

### Substrate spectra of CalDOX and LepDOX

For the investigation of the substrate spectra, CalDOX was pre-examined for saturated fatty acids (SFAs) ranging from caproic acid (C6:0) to stearic acid (C18:0) in the absence or presence of 1% (v/v) Triton X-100 (Supplemental Fig. S4). Detergents have been often used to increase the solubility of hydrophobic compounds (Hammer et al. 2020; Kaehne et al. 2011) and are helpful to offset the solubility effect of substrates with different hydrophobicities. With Triton X-100, the oxygenase activity for all the tested substrates substantially increased, and this effect became more prominent with substrates with higher carbon-chain length. Besides the activity, substrate preference of the enzyme was changed with the supplementation of the detergent as described below.

**Fig. 4:**
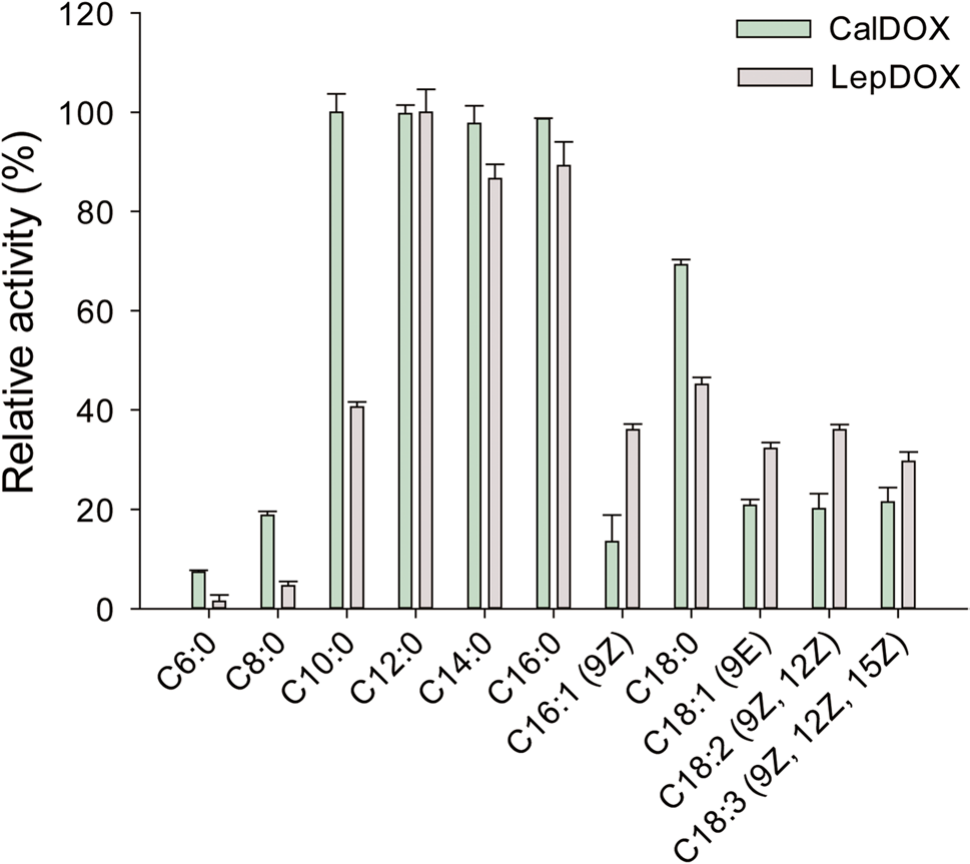
Substrate spectra of purified CalDOX and LepDOX in the presence of Triton X-100 (1%, v/v). Various fatty acids were used as the substrates: caproic acid (C6:0), caprylic acid (C8:0), capric acid (C10:0), lauric acid (C12:0), myristic acid (C14:0), palmitic acid (C16:0), and stearic acid (C18:0) as saturated substrates and palmitoleic acid (C16:1 (9*Z*)), elaidic acid (C18:1 (9*E*)), linoleic acid (C18:2 (9*Z*, 12*Z*)), and α-linolenic acid (C18:3 (9*Z*, 12*Z*, 15*Z*)) as unsaturated substrates. Each substrate at 2-mM was incubated with purified CalDOX (0.25 or 1 μg) or LepDOX (0.07 or 0.55 μg) at 25 °C and pH 7 in the presence of 1% (v/v) Triton X-100. Enzyme activities were determined at the linear phase (1 to 5 min depending on the substrate) in oxygen depletion kinetics. Relative activities (%) for each substrate represent the activities relative to the maximal values, set as 100%, which correspond to the values with C12:0 for both enzymes. Specific activities of CalDOX and LepDOX for C12:0 were 113 and 497 U/mg, respectively. Data represent means ± standard deviations of at least three replicates

Next, the substrate spectra of CalDOX and LepDOX were evaluated in the presence of Triton X-100, further including the valuable unsaturated fatty acids (USFAs), such as palmitoleic acid (C16:1 (9*Z*)), elaidic acid (C18:1 (9*E*)), linoleic acid (C18:2 (9*Z*, 12*Z*)), and α-linolenic acid (C18:3 (9*Z*, 12*Z*, 15*Z*)) (Fig. 4). Relative activities (RA; %) for each substrate represent the activities relative to the maximal values, set to 100%, which correspond to the values with C12:0 for both enzymes. The behavior of the two α-DOXs shown for SFAs was generally similar in that both enzymes exhibited the maximal and near-maximal activities for lauric acid (C12:0), myristic acid (C14:0), and palmitic acid (C16:0) (> 80% of RA). For shorter-chain SFAs, only CalDOX showed a good preference for C10:0 (100% of RA) and observable activities for C6:0 and C8:0 (7.4 and 18.8% of RA, respectively), whereas LepDOX showed a moderate activity for C10:0 (40.6% of RA) and no detectable activities for C6:0 and C8:0. This implies that CalDOX better accepts shorter substrates and has a broader substrate scope. When examined for USFAs, the enzyme activities were lower than those of SFAs with the same chain length (i.e., C16:0 and C18:0). One plausible reason is that USFAs are less solubilized by Triton X-100 than SFAs (Ahyayauch et al. 2006). Furthermore, there was no big difference in the activity among the USFAs. Whereas the introduction of a double bond reduced activities, the number or configuration of double bonds in USFAs may not be crucial for the activity of CalDOX and LepDOX.

To confirm the functionality of cyanobacterial α-DOXs, products were analyzed by GC-FID (Supplemental Fig. S5). Both purified CalDOX and LepDOX synthesized the expected tridecanal, the one carbon-reduced aldehyde, as the major product from myristic acid.

### Effects of temperature, pH, and metal ions

**Fig. 5:**
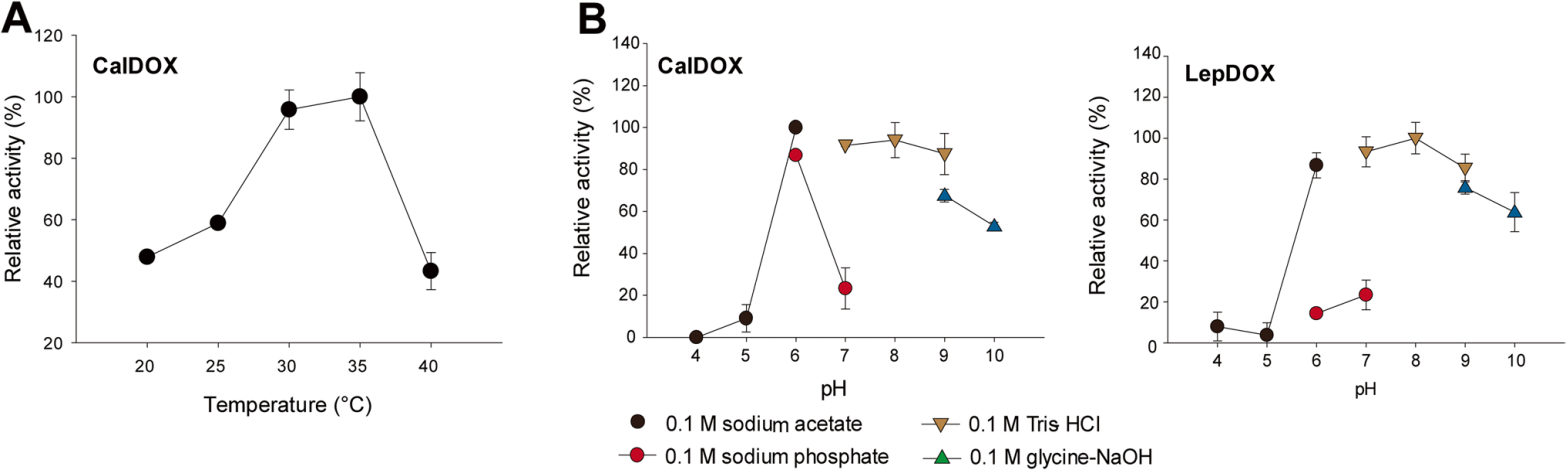
Effect of **a** temperature on CalDOX activity and **b** pH on CalDOX and LepDOX activities. **a** Enzyme reactions were performed with 2-mM of myristic acid without Triton X-100 for 20 min, initiated by 3.3 μg of purified CalDOX under temperature conditions ranging from 20 to 40 °C with the interval of 5 °C at pH 7 (0.1-M Tris–HCl) and 1000 rpm. Activity was determined by GC analysis. **b** Reactions were performed with 2-mM myristic acid with Triton X-100 (1%, v/v), initiated by purified enzymes (0.1 or 1 μg) at 25 °C under various pH values: 0.1-M sodium acetate (pH 4 to 6), 0.1-M sodium phosphate (pH 6 to 7), 0.1-M Tris–HCl (pH 7 to 9), and 0.1-M glycine–NaOH (pH 9 to 10). Activity was determined by the oxygen depletion assay at the initial time phase

The temperature optimum was observed by measuring fatty aldehydes produced from enzymatic reactions based on GC-FID analysis. Since Triton X-100 may interfere with the extraction of products, it was not included in these enzymatic reactions. In this study, the temperature optimum was investigated only for CalDOX since the activity of LepDOX was too low to obtain reliable GC peaks for quantification in the absence of detergents (Supplemental Fig. S5b). The maximum activity of CalDOX was found at 30 and 35 °C with no significant difference (*p* > 0.05) (Fig. 5a). The enzyme activity sharply decreased at temperatures lower than 30 and above 40 °C, showing less than 60% of RA. The original organism of CalDOX, *C. parietina*, was isolated from lake water, and the genus *Calothrix* is known to grow in the range of 25 and 40 °C (Issa 1999), which is in accordance with our results.

The effect of pH was studied at the pH range of 4–10 (Fig. 5b). Four buffering systems (0.1 M) were used: sodium acetate, sodium phosphate, Tris–HCl, and glycine–NaOH. Both enzymes showed a broad plateau of maximum and near-maximum activity (> 80% of RA) under slightly acidic to strongly alkaline conditions (i.e., pH 6 to 9). Little activity was retained at very acidic conditions, such as pH 4 and 5. The activities of CalDOX and LepDOX were dramatically lower in sodium phosphate buffers at pH 6 or 7 compared to the same pHs in acetate or Tris buffers. One possible reason might be that a buffer component (i.e., phosphate) acted as the inhibitor for the enzyme. Although also a universal buffer system using Davies buffer (Davies 1959) was carried out to avoid the effect of an individual buffer component, activity was shown only at pH 7, which was, however, tenfold lower than the activity at pH 7 with Tris–HCl (Supplemental Fig. S6). Furthermore, PIPES buffer (pH 6 and 7) also strongly inhibited the CalDOX activity (data not shown). Previous literature about AtDOX also revealed that its activity was sensitive to the buffering components including PIPES, HEPES, and MOPS (Liu et al. 2006). Therefore, Tris buffer at pH 7 was used for the routine in vitro enzyme assays in this study.

Previously, metal ions were reported as inhibitors for plant α-DOXs. The oxygenase activities of CalDOX and LepDOX were also studied in the presence of 1-mM of various metal ions during fatty acid conversion (Table 1). All of the tested metal ions except for Mg^2+^ significantly reduced the relative activities of both enzymes.

**Table 1:** Effect of metal ions

Metal ion (1-mM)	Relative enzyme activity^b^ (%)	
	CalDOX	LepDOX
Control^a^	100.0 ± 7.5	100.0 ± 5.6
MgCl_2_	93.6 ± 3.9	100.3 ± 1.8
ZnCl_2_	37.3 ± 5.2	41.5 ± 6.7
CuCl_2_	29.2 ± 1.9	36.1 ± 0.6
CoCl_2_	62.8 ± 7.1	87.0 ± 7.0
FeCl_3_	73.7 ± 3.2	89.1 ± 1.4
MnSO_4_	55.0 ± 2.8	67.2 ± 1.3
CaCl_2_	76.0 ± 6.3	67.7 ± 3.3

^a^Control represents the enzyme reaction in the absence of metal ions

^b^Enzyme activities were determined by the oxygen depletion assay with 2-mM myristic acid in a buffer containing 1% (v/v) Triton X-100 by incubating with purified CalDOX or LepDOX (0.1 to 1 μg) at 25 °C and pH 7 (0.1 M Tris–HCl) in the presence of 1-mM of various metal ions

### Whole-cell biotransformations for the synthesis of fatty aldehydes

**Fig. 6:**
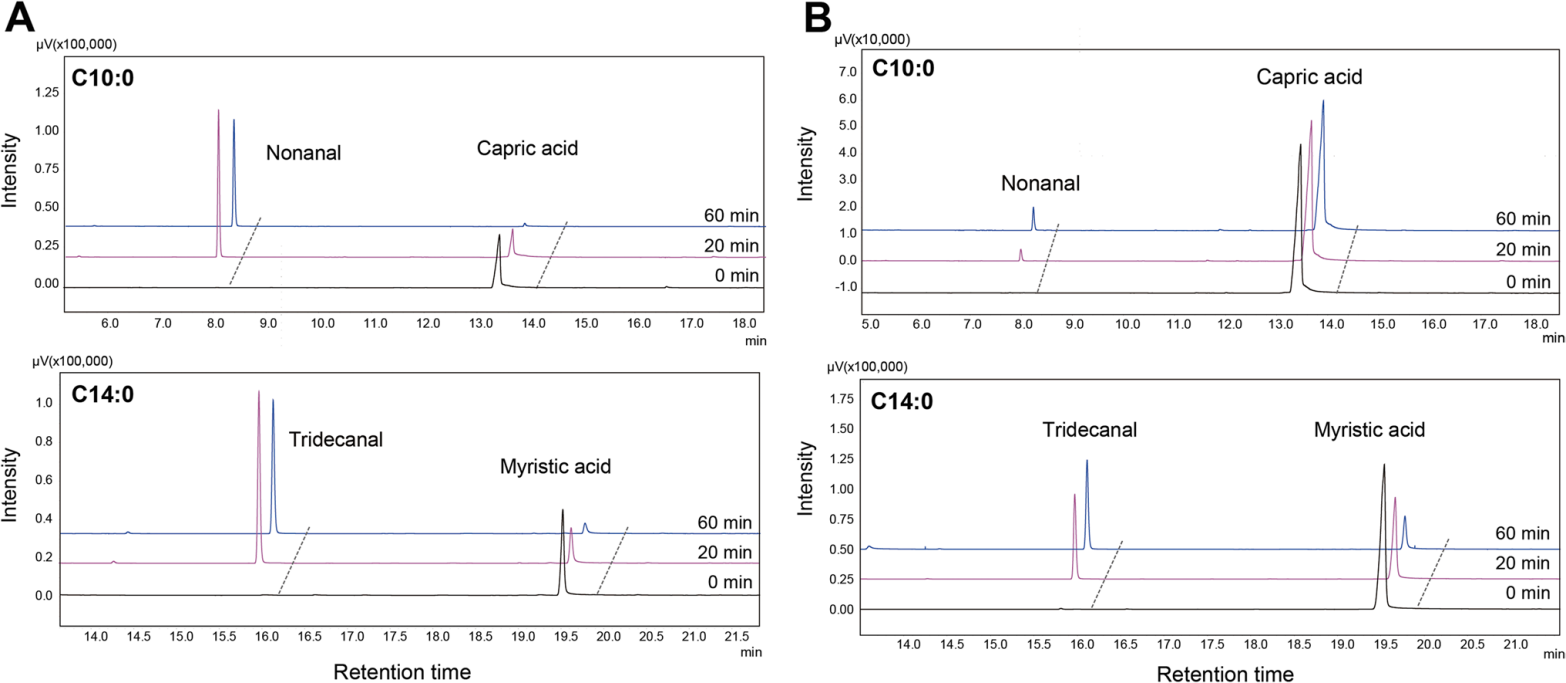
Profiles of substrate and product of the *E. coli* biotransformation analyzed by GC. Whole-cell biotransformations were conducted with *E. coli* resting cells at 12.5 g wet weight cell/L, which were incubated in the buffer containing 200-mM potassium phosphate with 50-mM NaCl (pH 7.4) at 35 °C and 1000 rpm with a reaction volume of 0.3 mL. The reaction was initiated by the addition of 5-mM of **a** capric or **b** myristic acid from 100-mM of a DMSO stock solution. After incubating for the indicated times, the biotransformations were stopped by the addition of 2-M HCl (30 μL), and the samples were extracted three times with an equal volume of ethyl acetate

In this study, the catalytic potential of whole *E. coli* resting cells expressing CalDOX or LepDOX was evaluated by exogenous supplementation of two different fatty acids, capric and myristic acids in 0.2-M potassium phosphate buffer with 50-mM NaCl (pH 7.4). Both enzymes were able to produce the expected C_n-1_ aldehydes, nonanal and tridecanal, respectively, as the major products by conversion of capric and myristic acids in *E. coli* resting cells (Fig. 6). In contrast, no corresponding product formation was observed with control *E. coli* cells containing an empty vector (data not shown). Meanwhile, neither C_n-1_ alcohols nor C_n-1_ FAs, produced by over-reduction or re-oxidation of fatty aldehydes by endogenous fatty aldehyde reductases or oxidases, respectively, were detected in this study although they were often produced by whole cells containing fatty aldehyde-producing α-DOX or CAR in previous studies (Foo et al. 2017; Maurer et al. 2019). The catalytic performance of CalDOX was found to be superior to that of LepDOX in the whole-cell *E. coli* system (Fig. 6 and Supplemental Fig. S7). For both substrates, CalDOX achieved full conversion in 20 or 40 min from 5-mM of capric or myristic acid, respectively. Both, conversion rate and yield for LepDOX-catalyzed reactions were much lower compared to CalDOX. The higher catalytic performance of CalDOX can be explained, for example, by the more intense red color of CalDOX-harboring cells, indicating a higher heme incorporation when expressed in *E. coli* (Fig. 2). The catalytic performance of LepDOX was particularly poor for the shorter capric acid showing only conversion of 4.2–8.6% (mol/mol). However, the enzyme displayed a substantially higher conversion for myristic acid (i.e., ~ 50%, mol/mol). These observations are in line with our in vitro substrate spectrum results, which revealed CalDOX had activities toward C10:0 and C14:0 with comparably high levels, whereas the acceptance of C10:0 by LepDOX was reduced (Fig. 4). In case of CalDOX, the amount of fatty aldehyde already synthesized decreased with the incubation time after achieving the full conversion (Supplemental Fig. S7). As no detectable peak was observed in chromatograms that could arise from fatty aldehyde degradation in the aqueous system or from cellular metabolism, evaporation of the fatty aldehydes might have contributed to the loss in mass.

## Discussion

Although most α-DOXs studied so far are from plants (Kaehne et al. 2011; Koeduka et al. 2005; Liu et al. 2006), the existence of α-DOX enzymes in non-plant organisms should not be excluded considering the recent discovery of a cyanobacterial α-DOX (Hammer et al. 2020). However, the complex phylogeny across taxa had not been examined yet. Our phylogenetic analysis revealed that α-DOXs are widely distributed among the various organisms other than plants. Such an unexpected diversity suggests the possible existence of the equivalent α-oxidation mechanism in non-plant organisms to that in plants, providing a novel hint for the occurrence of α-oxidation systems in other taxa. This is meaningful since identical fatty acid metabolisms have not been reported in non-plant taxa to date. Furthermore, the distribution of α-DOX in primitive bacteria as well as complex plants indicates an evolutionary origin back to the period prior to the appearance of plants.

**Fig. 7:**
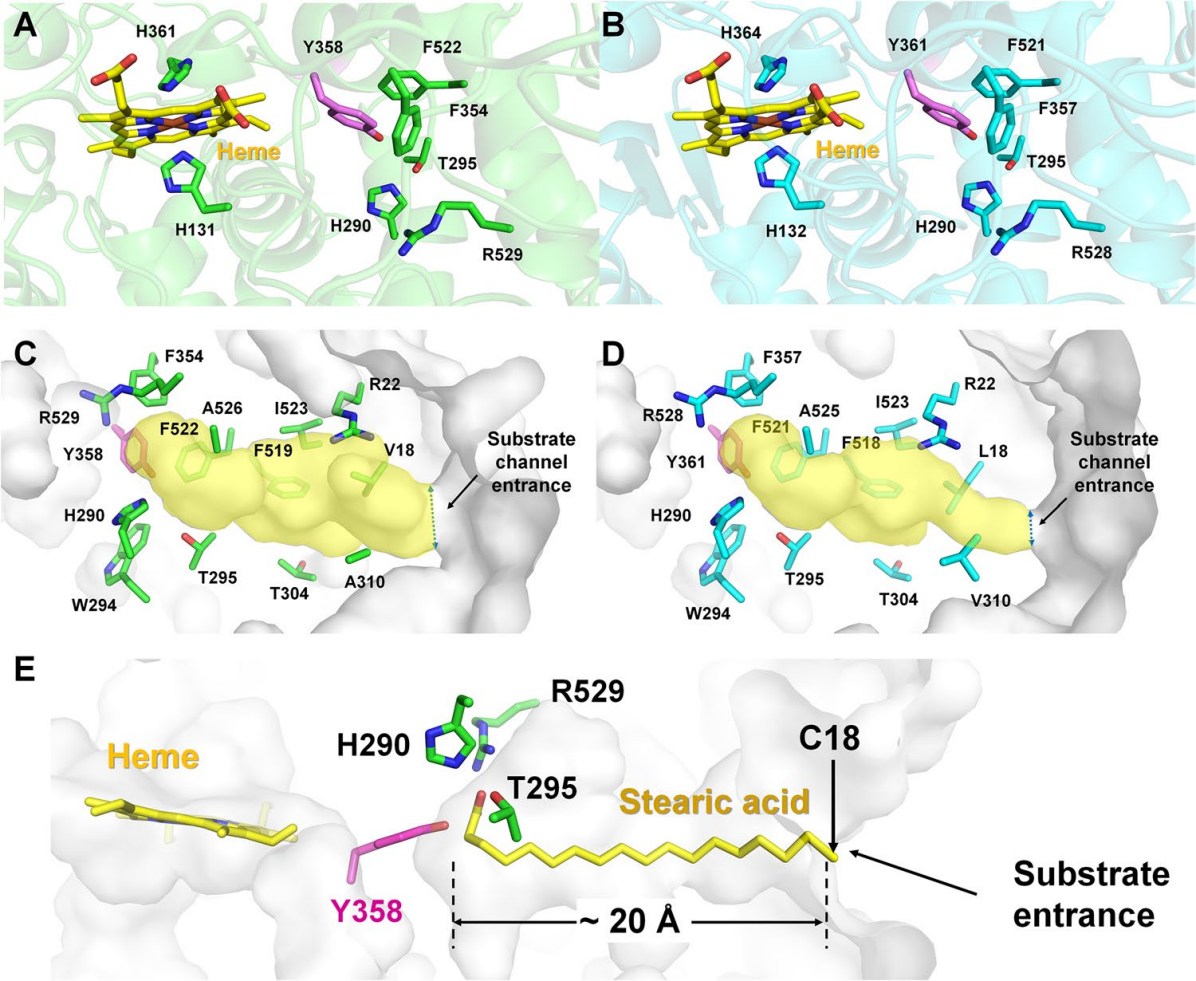
Homology models of CalDOX and LepDOX. Active site cleft present in the structural models of **a** CalDOX and **b** LepDOX in a cartoon representation. The functionally important residues, such as heme ligands (H131 and H361 in CalDOX and H132 and H364 in LepDOX), catalytic residues (Y358 in CalDOX and Y361 in LepDOX), and some fatty acid–interacting sites residing close to the catalytic residue (H290, T295, R529, F354, and F522 in CalDOX and H290, T295, R528, F521, and F357 in LepDOX) are shown as sticks. The surface representation of **c** CalDOX and **d** LepDOX homology models reveal 13 potential substrate-binding recognition sites in the binding cleft (V18, R22, H290, W294, T295, T304, A310, F519, F522, F354, I523, A526, and R529 in CalDOX and L18, R22, H290, W294, T295, T304, V310, F357, F518, F521, I523, A525, and R528 in LepDOX) of the enzymes. **e** The model structure of CalDOX in complex with stearic acid. The catalytic tyrosine is shown in violet

For plant α-DOXs, heme is the key constituent involved in their oxygenase activity. The recombinant CalDOX and LepDOX from cyanobacteria expressed in *E. coli* were also found to have heme-dependent oxygenase activity in this study. Our homology model analysis revealed that the key residues involved in the oxygenase activity of plant α-DOXs are also obviously conserved in CalDOX and LepDOX (Fig. 7a, b and Supplemental Fig. S8), supporting this phenomenon. To be specific, in the α-DOX–catalyzed reactions, the radical form of tyrosine (Y379 of OsaDOX and Y386 of AthDOX), which is generated by the activated heme moiety, is responsible for the oxygen incorporation to the α-carbon of the fatty acid (Mukherjee et al. 2011). This catalytic tyrosine is stabilized by π-π stacking interactions by phenylalanine residues (F375 and F552 for OsaDOX and F382 and F559 for AthDOX) (Supplemental Fig. S8) (Goulah et al. 2013; Zhu et al. 2013). In OsaDOX, the iron atom in the heme molecule has a distance from the catalytic tyrosine residue of ~ 13.0 Å, and the H157 and H382 residues as the distal and proximal ligands are coordinated with the iron atom (Supplemental Fig. S8a). The catalytic tyrosine and two phenylalanine residues in our model structures of CalDOX and LepDOX were revealed sequentially and positionally equivalent to plant DOXs (Fig. 7 and Supplemental S8). Furthermore, the two histidine residues (H131 and H361 for CalDOX, and H132 and H364 for LepDOX) were also at structurally identical positions of OsaDOX with respect to the heme ligands (Fig. 7a and b).

Native plant α-DOXs are considered to be monotopic membrane proteins (Goulah et al. 2013), and the recombinant α-DOXs expressed in *E. coli* are often treated with several kinds of detergents in cell lysis or purification (Hammer et al. 2020; Liu et al. 2006). In our study with cyanobacterial α-DOXs, the treatment of Triton X-100 during cell lysis was effective for enhancing the extraction yield and purified enzyme’s heme content and activity, indicating recombinant cyanobacterial α-DOXs also possibly interact with the lipid membranes of *E. coli*. *N*-terminal amphipathic α-helices of plant α-DOXs are known to be associated with membrane lipid droplets in plants (Bannenberg et al. 2009; Goulah et al. 2013). These α-helices play a role in the connection of the amphipathic membrane and catalytic site residing in the aqueous cellular compartment. Localization of α-DOXs close to fatty substrates can make it easier to extract lipid molecules, which are then guided through hydrophobic channels to the active site. In spite of lacking information on cyanobacterial fatty acid α-oxidation systems, it should be mentioned that cyanobacterial cells also contain lipid droplets (Lundquist et al. 2020), which suggests an idea for the possible localization of cyanobacterial α-DOXs in a native environment.

The substrate scope of most plant α-DOXs has been only investigated with fatty acids longer than 14 carbon atoms, and the comparative information on shorter substrates is lacking (Koszelak-Rosenblum et al. 2008; Liu et al. 2006). The previously known α-DOX from cyanobacteria, CsDOX, was revealed to have a different substrate spectrum from rice OsaDOX, in which CsDOX showed a better performance on shorter-chain substrates (< C16) than OsaDOX (Hammer et al. 2020). Particularly, CsDOX preferred C12:0 and C14:0, and then, activity was gradually reduced with a decreasing carbon length of substrate, which was similar to the behavior of LepDOX. In contrast, CalDOX even accepted C10:0 as the best substrate in addition to C12:0 and C14:0. Our whole-cell analysis also proved the superior capability of CalDOX for C10:0. Collectively, cyanobacterial α-DOXs were found to have a better acceptance of shorter substrates and a broader substrate scope than plant α-DOXs. Keeping in mind that fatty aldehydes with chain length from 8 to 13 are often used as flavor and fragrance compounds, such properties of cyanobacterial α-DOXs are advantageous when applied for industrial applications. In the complex structure of OsaDOX with palmitic acid (PA), 13 residues are positioned in the fatty acid binding channel, which stretches ~ 20 Å from the substrate entrance to the catalytic tyrosine. These were revealed as the substrate recognition sites (L53, R57, H311, W315, T316, M325, A331, F379, F549, F552, I553, A556, and R559) (Zhu et al. 2013). Among them, H311 and R559 are close to the catalytic tyrosine, and they have ionic interactions with the carboxylate group of fatty acid, and T316 plays a role in α-carbon positioning (Liu et al. 2006). These three residues are conserved in CalDOX and LepDOX (Fig. 7 and Supplemental S8). The model structures of CalDOX and LepDOX have a similar depth of substrate binding channel compared to plant α-DOXs (~ 20 Å) (Fig. 7e). However, the hydrophobic residues aligning in the substrate binding channel were not fully conserved among the analyzed α-DOXs (Supplemental Fig. S8c). For example, M325 of OsaDOX interacting with the C8 atom of PA was replaced by T304 in CalDOX and LepDOX. L53 of OsaDOX interacting with C12 atom of PA was replaced by V18 in CalDOX. Accordingly, the width and shape of the entrance site of the substrate binding channel were different in the structure of the CalDOX and LepDOX models (Fig. 7c and d). Such a variation in some substrate-interacting residues may well explain the different substrate spectrum of α-DOXs (Hammer et al. 2020; Koszelak-Rosenblum et al. 2008; Liu et al. 2006). The docking model of CalDOX with stearic acid reveals a substrate binding channel of CalDOX with a depth of ~ 20 Å, which is able to accept the SFA with 18 carbons at most (Fig. 7e). This implies the region beyond C18 in aliphatic fatty acids might not have a direct effect on substrate binding and selectivity of α-DOXs.

It should be noted that biochemical factors such as temperature, pH, and metal ions investigated here highly affect the nature of the fatty acids as well as of the enzyme. For example, fatty acid solubility and structure can be influenced by the pH condition. Under alkaline conditions, a fatty acid is more solubilized and forms micelle structures. In contrast, fatty acids are less solubilized and form crystal structures under acidic condition. Accordingly, the pH profiles obtained here not only attribute to the effect of pH on the biochemical properties of enzyme, but also to those of the substrates. Moreover, metal ions can form an insoluble complex with fatty acids via their carboxyl group (Chen et al. 2011), reducing the amount of available substrate for enzymes. In this context, the lower activities of α-DOXs shown by metal ions in this study may not be solely the consequence of the direct effect of metals on the enzyme itself. Therefore, it would be also of interest in future studies to evaluate the direct effect of metal ions on the enzyme’s properties without fatty acid substrate.

While the activities of purified enzymes were highly inhibited at high concentrations (0.1 M) of phosphate buffer at pH 7, whole-cell catalysis was done using 0.2-M potassium phosphate (pH 7.4) as the buffering agent in this study. It is well-known phenomenon that in vitro and in vivo systems differently affect the enzyme’s behaviors (de Carvalho 2017; Kratzer et al. 2011, 2015). In in vivo systems, intracellular enzymes are protected from the external environment by the cellular membrane and strategies to keep the intracellular environment homeostatic, whereas isolated free enzymes are susceptible to inactivation (Baker-Austin and Dopson 2007; de Carvalho 2017). Accordingly, there are frequent cases that the behavior of enzymes present within the cell is not directly affected by the extracellular condition (e.g., intracellular enzymes from extremophiles like halophiles and acidophiles) (de Carvalho 2017; Oren 2008). Similarly, inorganic phosphate is the essential compound for life including *E. coli* as the component of lipid membrane, building block of DNA or RNA, and energy source in the form of ATP. Accordingly, intracellular phosphate concentration is maintained at optimal levels in the range of 1 and 10-mM (that is, homeostasis) for *E. coli* by sophisticated uptake by transporters and control of metabolic reactions (Shulman et al. 1979; Xavier et al. 1995). This may well explain why CalDOX and LepDOX performed well in the cellular environment with phosphate buffer. There are several advantages when applying α-DOX in whole-cell biotransformations. As a potentially membrane-associated protein, α-DOXs may exhibit their catalytic activities better in a living cell with an intact membrane. Additionally, unlike CARs, demanding expensive cofactors, such as NADPH and ATP, and an additional of enzymes for posttranslational modification (i.e., phosphopantetheinyl transferases) (Akhtar et al. 2013; Wu et al. 2021), only aeration is sufficient to supply of oxygen when utilizing α-DOX in a whole-cell system. Although both enzymes produced the expected C_n-1_ aldehydes as the major products, C_n-1_ alcohols or C_n-1_ fatty acids, the possible side products or new substrates for α-DOX, respectively, were not detected in this study. The aldehyde-reducing capability in whole cells is dependent on the cell physiology associated with the intracellular redox state (Maurer et al. 2019). In a previous report, compared to CAR-expressing cells with a higher NADH/NAD^+^ ratio reflecting more reductive environment and reduced growth rate, α-DOX–expressing cells had a significantly lower amount of alcohol side product (Maurer et al. 2019). This may be the reason for the lack of observance of fatty alcohols in this study. The limited substrate specificity of fatty aldehyde dehydrogenases can be another reason. No detection of fatty acids with reduced carbon number could be due to their low yields as shown in the previous work in which the re-oxidized fatty acids accounted for only around 5% of the total products (Maurer et al. 2019).

It has been revealed throughout this study that heme-occupancy was the critical determinant for the catalytic performance of CalDOX and LepDOX both in vitro and in vivo. The different heme-incorporating capacity is probably because α-DOXs have different heme-binding environment. In OsaDOX, the heme molecule interacting with the H157 and H382 residues is further stabilized mainly by hydrophobic interaction from 23 amino acids (A149, I152, Q153, V156, H157, M160, D161, H162, N260, W262, T376, Y379, R380, M381, H382, I416, F453, L456, I470, L472, L475, R479, and R483) within 4 Å (Supplemental Fig. S8c). Model structures and sequence alignments show that these residues in the vicinity of the heme molecule are partially conserved among α-DOXs (Supplemental Fig. S8c), suggesting that α-DOXs may have different heme-binding affinities or selectivities (Liu et al. 2004). Another possible causal for incomplete incorporation of heme into CalDOX and LepDOX could be because the heme synthesized in *E. coli* is unnatural for cyanobacterial α-DOXs (Liu et al. 2004), which, therefore, may be enhanced by expressing the enzymes in different hosts.

In conclusion, we show that cyanobacterial α-DOXs are catalytically equivalent to plant α-DOXs but exhibit different substrate scope. Our in-depth molecular study of α-DOXs from non-plant organisms not only provides us with deeper understanding of this enzyme class but also facilitates the biosynthesis of fatty aldehyde–based aroma compounds in industry. To increase the industrial applicability of cyanobacterial α-DOXs, future studies need to be directed toward investigating their whole-cell catalytic performances at significantly higher substrate concentration than 5-mM.

## Supplementary Information

Below is the link to the electronic supplementary material.

Supplementary file1 (PDF 1575 KB)

## Data Availability

All data generated or analyzed during this study are included in this published article (and its supplementary materials file).

## References

[CR1] Ahyayauch H, Larijani B, Alonso A, Goñi FM (2006). Detergent solubilization of phosphatidylcholine bilayers in the fluid state: Influence of the acyl chain structure. Biochim Biophys Acta Biomembr.

[CR2] Akhtar MK, Turner NJ, Jones PR (2013). Carboxylic acid reductase is a versatile enzyme for the conversion of fatty acids into fuels and chemical commodities. Proc Natl Acad Sci U S A.

[CR3] Apweiler R, Attwood TK, Bairoch A, Bateman A, Birney E, Biswas M, Bucher P, Cerutti L, Corpet F, Croning MD, Durbin R, Falquet L, Fleischmann W, Gouzy J, Hermjakob H, Hulo N, Jonassen I, Kahn D, Kanapin A, Karavidopoulou Y, Lopez R, Marx B, Mulder NJ, Oinn TM, Pagni M, Servant F, Sigrist CJ, Zdobnov EM (2001). The InterPro database, an integrated documentation resource for protein families, domains and functional sites. Nucleic Acids Res.

[CR4] Baker-Austin C, Dopson M (2007). Life in acid: pH homeostasis in acidophiles. Trends Microbiol.

[CR5] Bannenberg G, Martínez M, Rodríguez MJ, López MA, Ponce de León I, Hamberg M, Castresana C (2009). Functional analysis of alpha-DOX2, an active alpha-dioxygenase critical for normal development in tomato plants. Plant Physiol.

[CR6] Chen L, Meng H, Jiang L, Wang S (2011). Fatty-acid-metal-ion complexes as multicolor superhydrophobic coating materials. Chem Asian J.

[CR7] Colas C, De Montellano PR (2004). Horseradish peroxidase mutants that autocatalytically modify their prosthetic heme group: insights into mammalian peroxidase heme-protein covalent bonds. J Biol Chem.

[CR8] Davies MT (1959). A universal buffer solution for use in ultra-violet spectrophotometry. Analyst.

[CR9] de Carvalho CC (2017). Whole cell biocatalysts: essential workers from nature to the industry. Microb Biotechnol.

[CR10] Edgar RC (2004). MUSCLE: multiple sequence alignment with high accuracy and high throughput. Nucleic Acids Res.

[CR11] Foo JL, Rasouliha BH, Susanto AV, Leong SSJ, Chang MW (2020). Engineering an alcohol-forming fatty acyl-CoA reductase for aldehyde and hydrocarbon biosynthesis in *Saccharomyces cerevisiae*. Front Bioeng Biotechnol.

[CR12] Foo JL, Susanto AV, Keasling JD, Leong SS, Chang MW (2017). Whole-cell biocatalytic and de novo production of alkanes from free fatty acids in *Saccharomyces cerevisiae*. Biotechnol Bioeng.

[CR13] Foster SP, Anderson KG (2019). Production and distribution of aldehyde and alcohol sex pheromone components in the pheromone gland of females of the moth *Chloridea virescens*. J Chem Ecol.

[CR14] Gaylord NG (1957). Reduction with complex metal hydrides. J Chem Educ.

[CR15] Gilbertson JR, Johnson RC, Gelman RA, Buffenmyer C (1972). Natural occurrence of free fatty aldehydes in bovine cardiac muscle. J Lipid Res.

[CR16] Gouet P, Courcelle E, Stuart DI, Métoz F (1999). ESPript: analysis of multiple sequence alignments in PostScript. Bioinformatics.

[CR17] Goulah CC, Zhu G, Koszelak-Rosenblum M, Malkowski MG (2013). The crystal structure of α-dioxygenase provides insight into diversity in the cyclooxygenase-peroxidase superfamily. Biochemistry.

[CR18] Hamberg M, Ponce de León I, Sanz A, Castresana C (2002). Fatty acid alpha-dioxygenases. Prostaglandins Other Lipid Mediat.

[CR19] Hammer AK, Albrecht F, Hahne F, Jordan P, Fraatz MA, Ley J, Geissler T, Schrader J, Zorn H, Buchhaupt M (2020). Biotechnological production of odor-active methyl-branched aldehydes by a novel α-dioxygenase from *Crocosphaera subtropica*. J Agr Food Chem.

[CR20] Hammer AK, Emrich NO, Ott J, Birk F, Fraatz MA, Ley JP, Geissler T, Bornscheuer UT, Zorn H (2021). Biotechnological production and sensory evaluation of ω1-unsaturated aldehydes. J Agr Food Chem.

[CR21] Hu Y, Zhu Z, Gradischnig D, Winkler M, Nielsen J, Siewers V (2020). Engineering carboxylic acid reductase for selective synthesis of medium-chain fatty alcohols in yeast. Proc Natl Acad Sci U S A.

[CR22] Huang Y, Niu B, Gao Y, Fu L, Li W (2010). CD-HIT Suite: a web server for clustering and comparing biological sequences. Bioinformatics.

[CR23] Hunter S, Apweiler R, Attwood TK, Bairoch A, Bateman A, Binns D, Bork P, Das U, Daugherty L, Duquenne L, Finn RD, Gough J, Haft D, Hulo N, Kahn D, Kelly E, Laugraud A, Letunic I, Lonsdale D, Lopez R, Madera M, Maslen J, McAnulla C, McDowall J, Mistry J, Mitchell A, Mulder N, Natale D, Orengo C, Quinn AF, Selengut JD, Sigrist CJA, Thimma M, Thomas PD, Valentin F, Wilson D, Wu CH, Yeats C (2009). InterPro: the integrative protein signature database. Nucleic Acids Res.

[CR24] Issa AA (1999). Antibiotic production by the cyanobacteria *Oscillatoria angustissima* and *Calothrix parietina*. Environ Toxicol Pharmacol.

[CR25] Kaehne F, Buchhaupt M, Schrader J (2011). A recombinant α-dioxygenase from rice to produce fatty aldehydes using *E. coli*. Appl Microbiol Biotechnol.

[CR26] Kalyaanamoorthy S, Minh BQ, Wong TKF, von Haeseler A, Jermiin LS (2017). ModelFinder: fast model selection for accurate phylogenetic estimates. Nat Methods.

[CR27] Koeduka T, Matsui K, Hasegawa M, Akakabe Y, Kajiwara T (2005). Rice fatty acid alpha-dioxygenase is induced by pathogen attack and heavy metal stress: activation through jasmonate signaling. J Plant Physiol.

[CR28] Koszelak-Rosenblum M, Krol AC, Simmons DM, Goulah CC, Wroblewski L, Malkowski MG (2008). His-311 and Arg-559 are key residues involved in fatty acid oxygenation in pathogen-inducible oxygenase. J Biol Chem.

[CR29] Kratzer R, Pukl M, Egger S, Vogl M, Brecker L, Nidetzky B (2011). Enzyme identification and development of a whole-cell biotransformation for asymmetric reduction of *o*-chloroacetophenone. Biotechnol Bioeng.

[CR30] Kratzer R, Woodley JM, Nidetzky B (2015). Rules for biocatalyst and reaction engineering to implement effective, NAD(P)H-dependent, whole cell bioreductions. Biotechnol Adv.

[CR31] Kunjapur AM, Prather KLJ (2015). Microbial engineering for aldehyde synthesis. Appl Environ Microbiol.

[CR32] Lehtinen T, Efimova E, Santala S, Santala V (2018). Improved fatty aldehyde and wax ester production by overexpression of fatty acyl-CoA reductases. Microb Cell Fact.

[CR33] Lehtinen T, Santala V, Santala S (2017) Twin-layer biosensor for real-time monitoring of alkane metabolism. FEMS Microbiol Lett 364(6). 10.1093/femsle/fnx053 10.1093/femsle/fnx053 28333269

[CR34] Liu K, Chen Q, Liu Y, Zhou X, Wang X (2012). Isolation and biological activities of decanal, linalool, valencene, and octanal from sweet orange oil. J Food Sci.

[CR35] Liu W, Rogge CE, Bambai B, Palmer G, Tsai AL, Kulmacz RJ (2004). Characterization of the heme environment in *Arabidopsis thaliana* fatty acid alpha-dioxygenase-1. J Biol Chem.

[CR36] Liu W, Wang LH, Fabian P, Hayashi Y, McGinley CM, van der Donk WA, Kulmacz RJ (2006). *Arabidopsis thaliana* fatty acid alpha-dioxygenase-1: evaluation of substrates, inhibitors and amino-terminal function. Plant Physiol Biochem.

[CR37] Lundquist PK, Shivaiah K-K, Espinoza-Corral R (2020). Lipid droplets throughout the evolutionary tree. Prog Lipid Res.

[CR38] Maurer S, Schewe H, Schrader J, Buchhaupt M (2019). Investigation of fatty aldehyde and alcohol synthesis from fatty acids by αDox- or CAR-expressing *Escherichia coli*. J Biotechnol.

[CR39] Mosblech A, Feussner I, Heilmann I (2009). Oxylipins: structurally diverse metabolites from fatty acid oxidation. Plant Physiol Biochem.

[CR40] Mukherjee A, Angeles-Boza AM, Huff GS, Roth JP (2011). Catalytic mechanism of a heme and tyrosyl radical-containing fatty acid α-(di)oxygenase. J Am Chem Soc.

[CR41] Noordermeer MA, Veldink GA, Vliegenthart JF (2001). Spectroscopic studies on the active site of hydroperoxide lyase; the influence of detergents on its conformation. FEBS Lett.

[CR42] Oren A (2008) Microbial life at high salt concentrations: phylogenetic and metabolic diversity. Saline Syst 4(2). 10.1186/1746-1448-4-2 10.1186/1746-1448-4-2 PMC2329653 18412960

[CR43] Reiser S, Somerville C (1997). Isolation of mutants of *Acinetobacter calcoaceticus* deficient in wax ester synthesis and complementation of one mutation with a gene encoding a fatty acyl coenzyme A reductase. J Bacteriol.

[CR44] Shulman RG, Brown TR, Ugurbil K, Ogawa S, Cohen SM, den Hollander JA (1979). Cellular applications of ^31^P and ^13^C nuclear magnetic resonance. Science.

[CR45] Sievers F, Higgins DG (2014) Clustal Omega, accurate alignment of very large numbers of sequences. In: Russell d. (eds) multiple sequence alignment methods. Methods in molecular biology (methods and protocols). Humana Press, Totowa, NJ 1079, pp105–116. 10.1007/978-1-62703-646-7_6 10.1007/978-1-62703-646-7_6 24170397

[CR46] Wu S, Snajdrova R, Moore JC, Baldenius K, Bornscheuer UT (2021). Biocatalysis: enzymatic synthesis for industrial applications. Angew Chem Int Ed.

[CR47] Xavier KB, Kossmann M, Santos H, Boos W (1995). Kinetic analysis by in vivo ^31^P nuclear magnetic resonance of internal P_i_ during the uptake of *sn*-glycerol-3-phosphate by the *pho* regulon-dependent Ugp system and the *glp* regulon-dependent GlpT system. J Bacteriol.

[CR48] Zhu G, Koszelak-Rosenblum M, Malkowski MG (2013). Crystal structures of α-dioxygenase from *Oryza sativa*: insights into substrate binding and activation by hydrogen peroxide. Protein Sci.

