# Supplementary material for: Two novel cyanobacterial α-dioxygenases for the biosynthesis of fatty aldehydes
Source: Appl Microbiol Biotechnol. 2021 Dec 9;106(1):197–210. doi: 10.1007/s00253-021-11724-x (PMC8720084; doi:10.1007/s00253-021-11724-x)
Supplement: Supplementary file 1 — Supplementary file1 (PDF 1575 KB) [file 253_2021_11724_MOESM1_ESM.pdf]

Supporting Information

Two novel cyanobacterial α-dioxygenases for the biosynthesis of fatty aldehydes

Running title: fatty aldehydes from fatty acids by cyanobacterial α-dioxygenases

In Jung Kim[1], Yannik Brack[1], Thomas Bayer[1], Uwe T. Bornscheuer[1,*]

[1]Department of Biotechnology and Enzyme Catalysis, Institute of Biochemistry, University of Greifswald, 17489, Greifswald, Germany

*E-mail: uwe.bornscheuer@uni-greifswald.de

A

ATGTTCGGCATTCCGTGGCATAAACTGCCGACCCCGCTGGGTCTGCTGAAAGTTGTGCAGTTCCGCGGTCGTCTGCGTAAAAATAAT
CTGCAGGATACCAGTCAGCTGGCCGATACCACCAAACTGCCGGAAACCATGAAATGCCCGTATCATAATTATCTGAAAGCACGCACC
CCGGATGGCAGTTATAATGATCTGAAACATCCGGAAATGGGCATGGTTGGCACCCGCTTCGGCCGTAATATTCCGCTGGAAGATGTTA
AAGTTGATGAAGCAAATCTGCTGACCCCGAATCCGCGCACCGTGAGCCGTATGCTGCTGGCCCGCGAAGAATTCCAGCCGGCAAC
CATTCTGAATCTGCTGGCAGCAGCCTGGATTCAGTTCCAGACCCATGATTGGTTCAGTCATGGTAATAATCAGCCGGATAATAAATTCC
AGATTCCGATTGATGCCGATGATAGCTGGCCGCAGGAACATCGCCCGATGGAAGTTAGCCGTACCCTGGAAGATCCGACCCGTAGT
GGCGATACCAAAAATCCGGCCACCTTCATTAATCATTGCACCCATTGGTGGGATGGTAGCCAGGTGTATGGCAATGATAAAAAAACCA
TTGAAGAGGTGCGCAGTCGTGTTAATGGCAAAATGCGTCTGGAAGAAGATGGCTTCCTGCCGCTGGATCCGAAACTGGGCATTGAT
AAAGTGGGCTTCCCGGGTGCATGGTGGGTGGGCCTGAGCATGCTGCATACCCTGTTCGTGAAAGAACATAATACCATCTGTGATCAT
CTGAGTAAACTGTATCCGGAATGGAAAGATGATGAACTGTTCGATCATGCACGTCTGATTAATGCAGCACTGATGGCCAAAATTCATAC
CGTGGAATGGACCCCGGCCATTCTGCCGCATCCGGTGACCGATATTGCACTGAATGCCAATTGGTGGGGTATTCTGGGCCAGCAGG
TTAAAGATACCGTGGGTCATATTGGCGATAGTGAACTGCTGAGCGGTATTATTGGCAGCCCGACCGATCATCATGCAGCCCCGTATTA
TCTGACCGAAGAATTCGTGAGCGTGTATCGTATGCATGCCCTGATTCCGGATGACTTCGACTTCTATAGTCTGAAAACCAGAAAACTG
CTGCATAAAAAAAGCTTCCCGGAAGTGGCAGGCAGTAAAACCAGAGCCTTCATGGAAGAAGTTCAGATGAGTGATCTGTTCTATAGC
TTCGGCATTAGCCATCCGGGTGCCGTGCGTCTGCATAATTATCCGAAATTCCTGCGTCAGCTGACCGGTGAACTGACCGGCGATCC
GAATGGTGCATTCGATCTGGCCGCAGTTGATATTCTGCGTGATCGTGAACGTGGCGTGCCGCGTTATAATCGCTTCCGTGAACTGAT
TGGTCGTGGTAAAGTTAAAAGCTTCGAAGAAATTACCAGCAATAAACAGTGGGCCAAAGAACTGCGTGAAGTGTATAATAATAACATTG
ATAGCGTGGATCTGATGGTTGGTCTGTATGCCGAAGATATTCCGGAAAAATTCGGCTTCAGTGATACCGCCTTCCGTGTGTTCATTCT
GATGGCCAGTCGTCGTCTGAAAAGCGATCGCTTCTTCACCAAAGATTATACCGCAGAAGTGTATACCCAGTTCGGTCTGGATTGGATT
GATCGCAATAATCTGGTGACCGTGCTGAAACGCCATCATCCGGAACTGGCACCGGTTCTGGTTAATGTGACCAATGGCTTCAAACCG
TGGAAATAA

B

ATGTTCGATAACTTCTGGCATAAACTGCCGACCCCGCTGGCACTGCTGCAGCTGCTGAAACTGCGCAATGATCTGCGTGAAAAAAAT
CTGCATGATACCAGTCAGCTGCCGGATAGCGGCGAACTGCCGAAACCGCAGCCGAGTAGTGATGGCAGTCATCTGACCGCACGCA
CCGCCGATGGTAGCTTCAATGATCTGCAGCAGCCGCAGATGGGCATGGCAGGTACCCGCTTCGGTCGCAATGTGGCACTGGGTGC
CGTTGAAGCCGAAGCAGTGCCGAAACTGATGACCCCGAATCCGCGTGAAGTTAGTCGTATTCTGATGACCCGTGATCGCTTCCAGC
CGGCAACCATGCTGAATCTGCTGGCAGCAGCATGGATTCAGTTCGAAAATCATGATTGGTTCAGTCATGGCGATAATGAACCGGATG
ATCAGCTGGAAATTCCGCTGGAACCGCATGATCCGTGGCCGGAAGAACATCGTCCGATGGTTGTTGGCAAAACCTTAGCAGATAAAA
GTCGTCCGGATGGCGCACGCCCGAAAACCTTCATTAATACCGTTACCCATTGGTGGGATGGCAGTCAGATCTATGGTAGCGATCCGG
AAACCGTGGATAAACTGCGTAGTCATGTTCATGGCAAACTGACCATTCAGGATGATGGTCTGCTGCCGGTTGATCCGGAAACAGGCC
TGGATGTTACCGGCTTCAATGATAATTGGTGGATTGGCCTGAGTATGCTGCATACCGTGTTCACCAAAGAACATAATGCAATCTGTGAT
CATCTGAAACAGGAATATCCGCATTGGAGTGATGATATTCTGTTCAATCATGCACGTCTGATTAATGCCGCACTGATGGCAAAAATTCA
TACCGTTGAATGGACCCCGGCCATTCTGCCGCTGCCGGTGACCGATATTGCACTGAATGTGAATTGGAGTGGCTTCCTGGGTGAAG
ATCTGAAACAGGTTCTGGGCAGCGTGGGCGAAGGTGAACTGACCGATCTGCTGAGTGGCATTGTTGGCAGCGAAACCGATCATCAT
ACCGCACCGTATTATCTGACCGAAGAATTCGCCAGCGTGTATCGTATGCATCCGCTGATTCCGGATGAACTGCAGTTCTATAGCCTGG
ATAATAATCAGCCGCTGCAGAGCGCAGACTTCTTCCAGATTAGCGGTAAACGTAGCCGTACCCTGCTGGAAAGCATTCCGATGCCGG
ATCTGTATTATAGCTTCGGTGTTGCCCATCCGGGCGAAATTAGTCTGCATAATTATCCGCGCAGCCTGCAGCAGCTGGTGCGTGATAA
TGGTGAAGTGTTCGATCTGGCCGCCGTGGATATTCTGCGCGATCGTGAACGTGGTGTGCCGCGCTATAATCGCTTCCGCGAACTGA
TTGGCCGCACCCGTGTTAAAAGCTTCGAAGAAATTAGTAGCAATCAGCAGTGGGTGGAAGAAATGCGTCGCGTGTATTATAATGATAT
TAATAGTGTGGACCTGATGGTTGGTCTGTTCGCAGAAGATAAACCGGAAGGCTTCGGCTTCAGCGATACCGCCTTCCGCGTGTTCAT
TCTGATGGCCAGTCGTCGTCTGAAAAGTGATCGCTTCTTCACCAAAGATTATCGCGCAGAAGTGTATACCCAGCTGGGCCTGGATTG
GATTGCCAATAATACCATGGTTACCGTGCTGCAGCGTCACTTCCCGACCCTGGCACCGGCACTGTATGATATTGATAATGCATTCAAA
CCGTGGCGTCGTATTGGTACCTAA

Fig. S1 Codon-optimized synthetic gene sequences for **a** CalDOX (GenBank accession no. MZ522724) and **b** LepDOX (GenBank accession no. MZ522725).

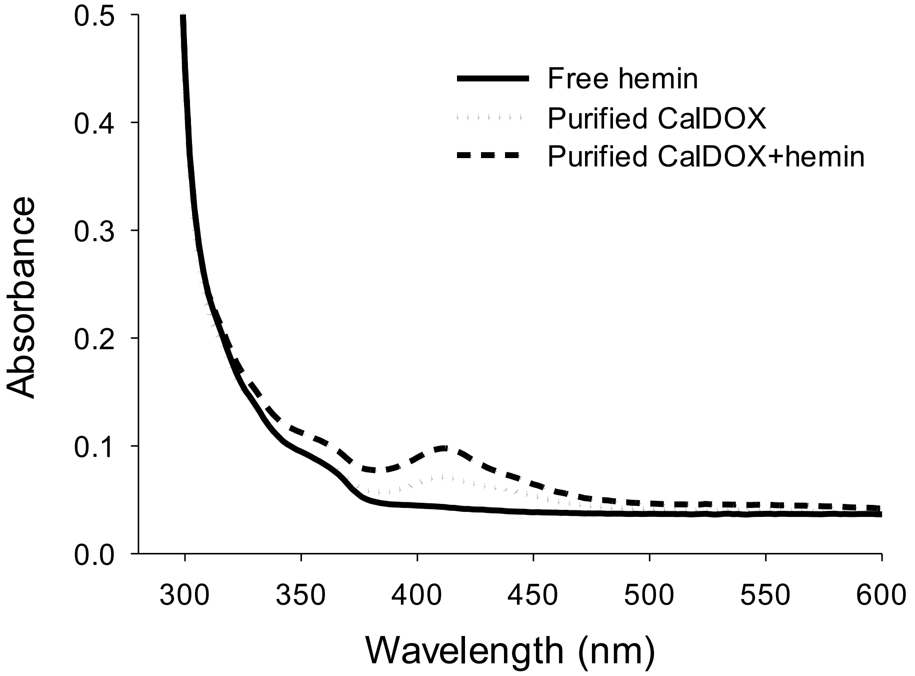

Fig. S2 Effect of hemin addition (4 μM) to purified CalDOX (4 μM) on Soret peak intensity.

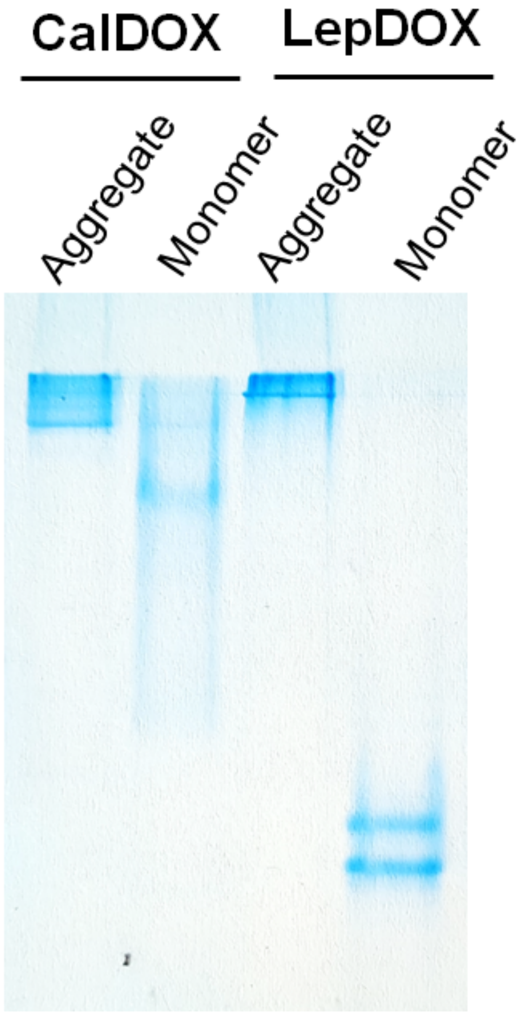

Fig. S3 Native gel analysis for aggregated and monomeric forms of CalDOX and LepDOX. Electrophoresis was run on 10% polyacrylamide gel in running buffer (25 mM Tris, 200 mM glycine, pH 8.4) at 100 V on ice. Gels and running buffers excluding SDS were prepared to maintain the native structure of proteins.

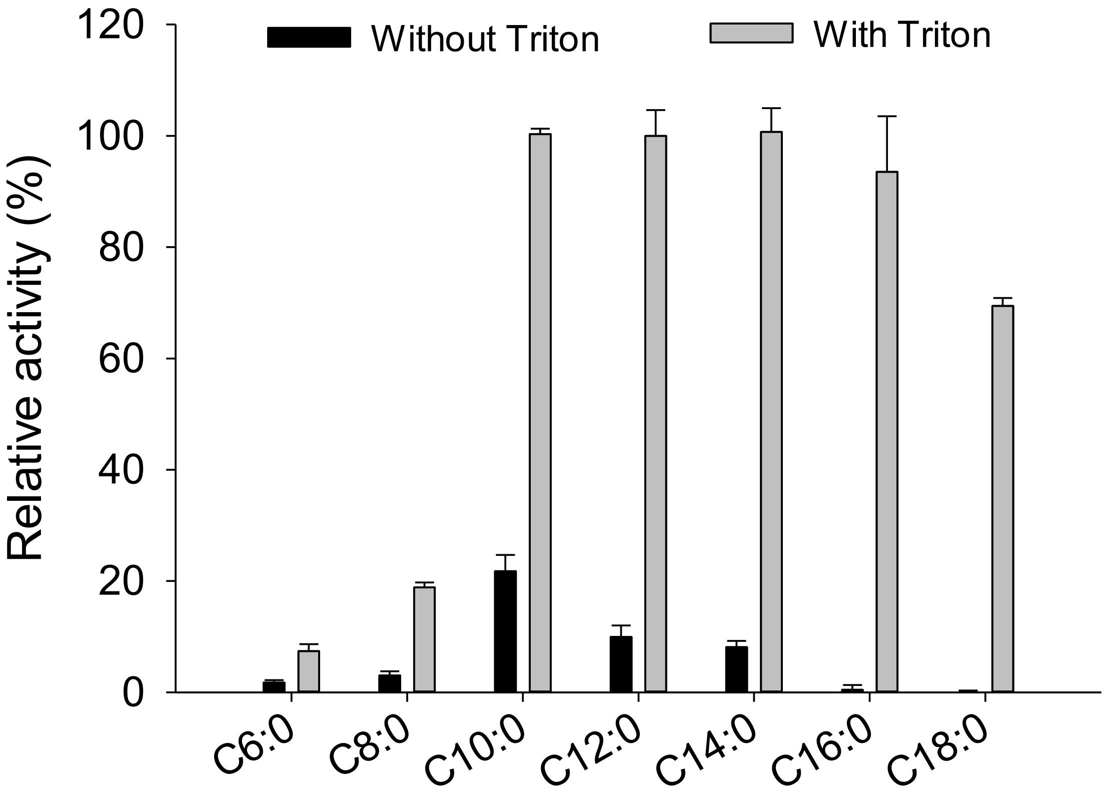

Fig. S4 Substrate spectrum of CalDOX in the absence or presence of 1% Triton X-100 (*v/v*) for saturated fatty acids ranging from caproic acid (C6:0) to stearic acid (C18:0). Relative activities (%) for each substrate represent the activities relative to the maximal values of C12:0 obtained in the presence of Triton X-100 (114 U/mg), set as 100%. Data represent means ± standard deviations of at least three replicates.

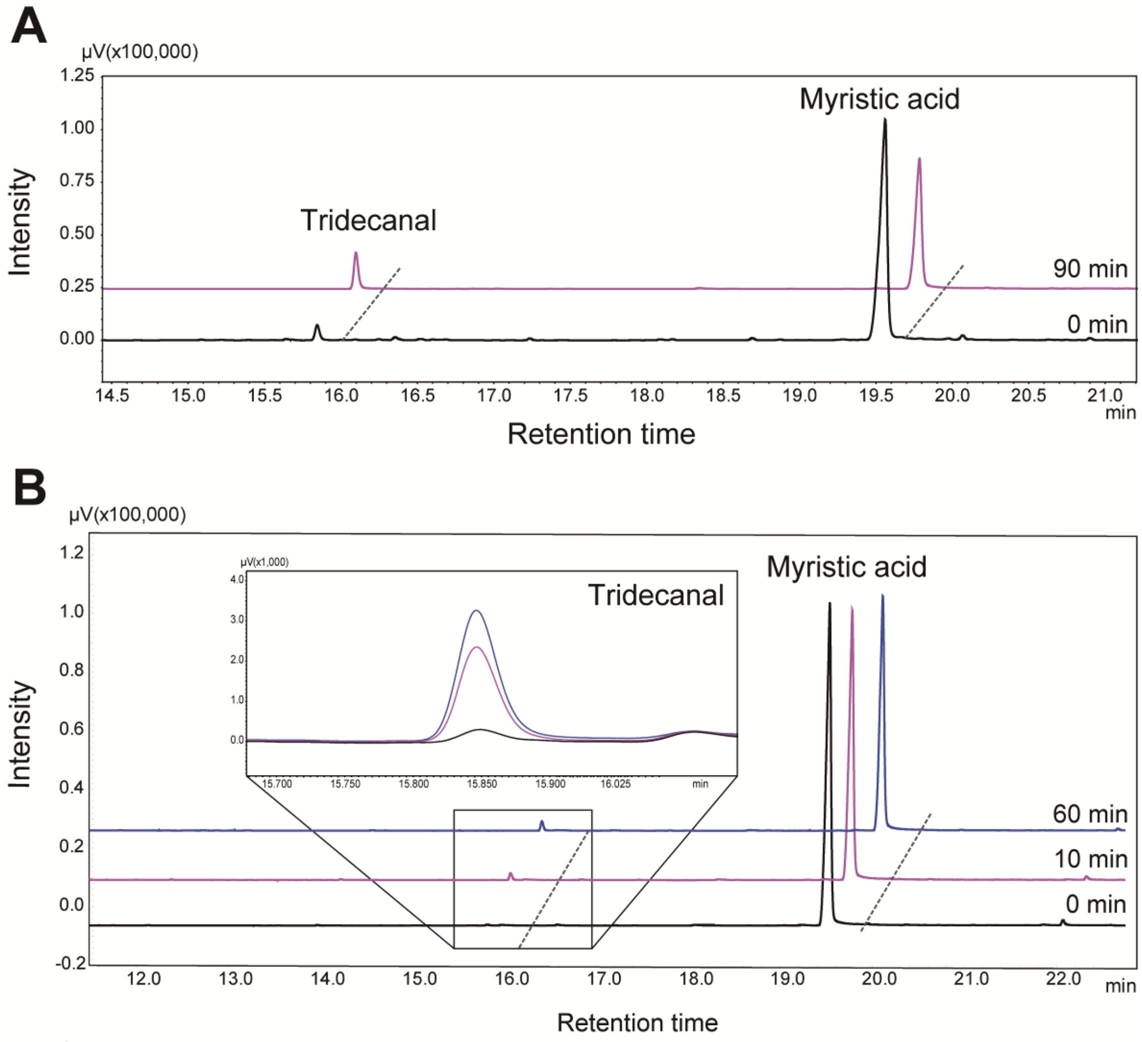

Fig. S5 Product identification of **a** CalDOX or **b** LepDOX using GC-FID analysis. The enzymatic reactions were performed by incubating 2 mM myristic acid with purified CalDOX (3.3 μg) or LepDOX (5 μg) at 35°C and pH 7 in 300 μL total reaction volume for indicated times with shaking (1,000 rpm). The insert in **b** shows the blow-up of peaks corresponding to the reaction product.

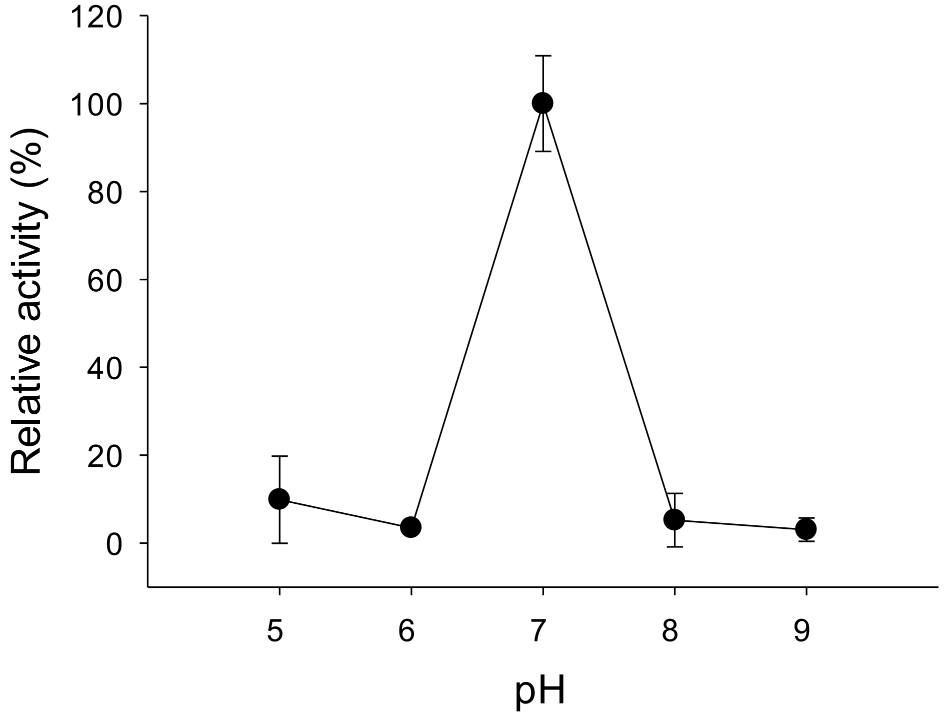

Fig. S6 Effect of pH on the CalDOX activity investigated using the one buffer system based on Davies buffer. Reactions were performed with 2 mM myristic acid with 1% (*v/v*) Triton X-100 at 25°C for 1 min, initiated by adding 1 µg of purified enzyme. The activity was determined by oxygen-depletion assay

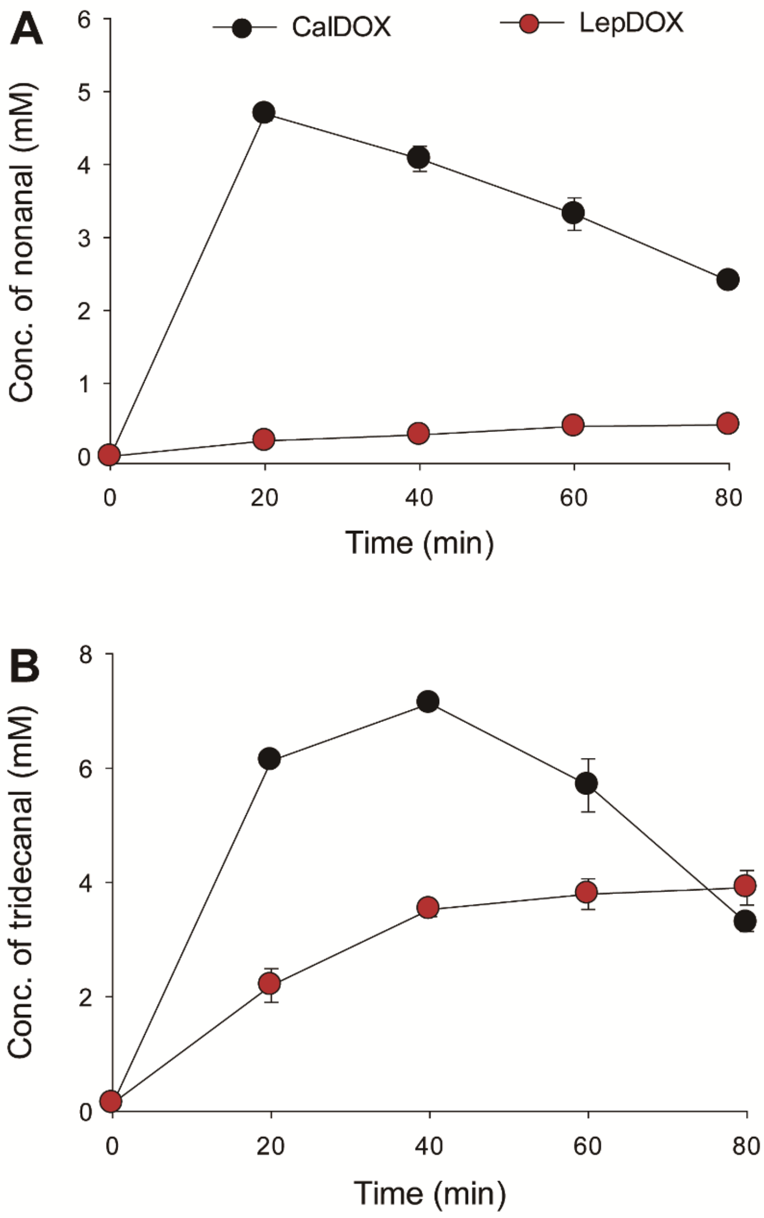

Fig. S7 Whole-cell application of CalDOX and LepDOX using *E. coli* resting cells with supplementation of 5 mM **a** capric acid or **b** myristic acid. The corresponding fatty aldehyde products were quantified based on the calibration curves using linear regression.

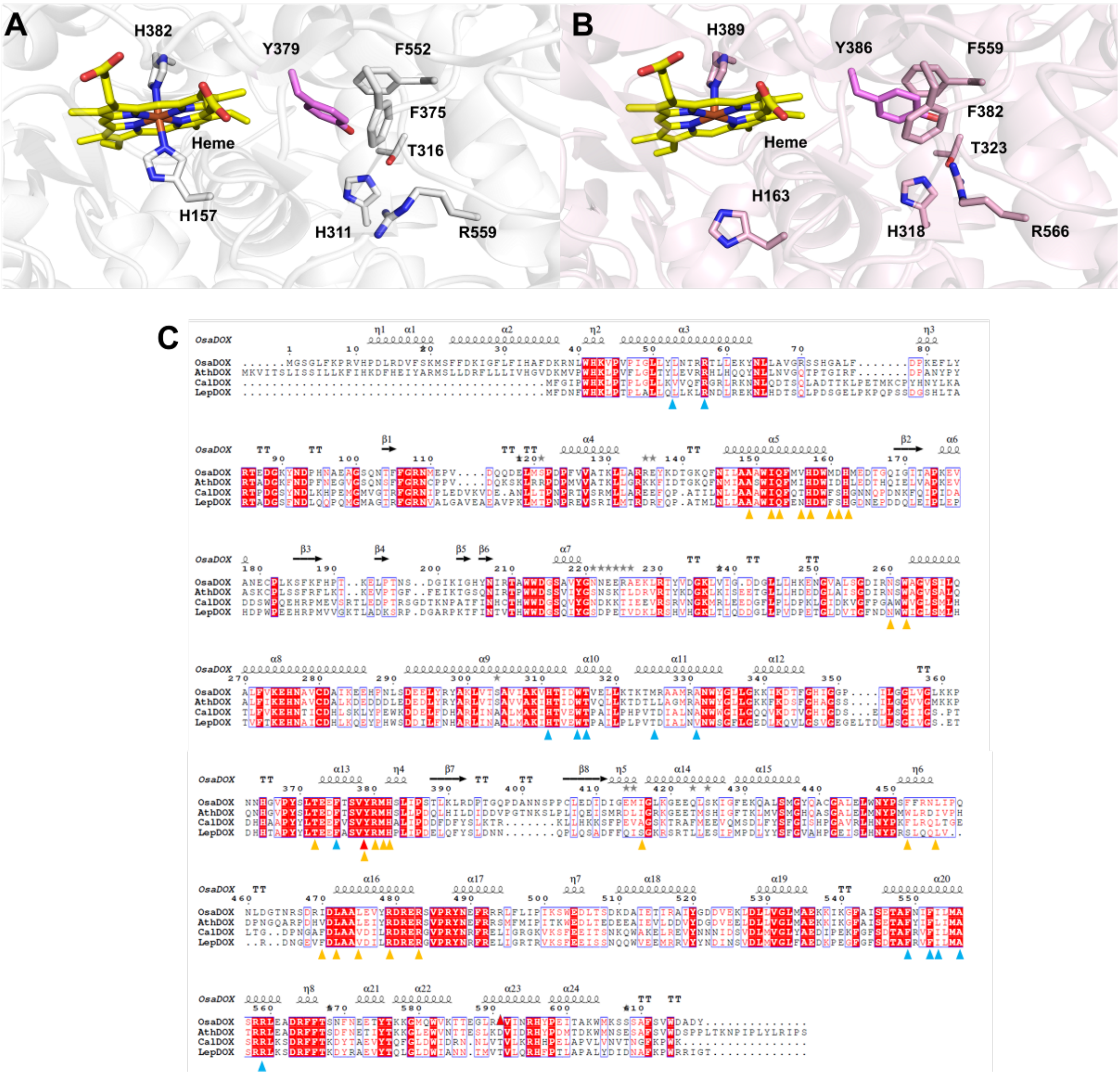

Fig. S8 Active site cleft in the crystal structures of **a** OsaDOX (PDB code: 4KVK) and **b** AthDOX (4HHS) in cartoon representation. The functionally important residues such as heme ligands (H157 and H382 in OsaDOX, H163 and H389 in AthDOX), catalytic residue (Y358 in OsaDOX and AthDOX), and some fatty acid-interacting sites close to the catalytic residue (H311, T316, R559, F375, and F552 in OsaDOX, H318, T323, F382, F559, and R566 in AthDOX) are shown as sticks. The catalytic tyrosine is shown in violet. **c** Multiple sequence alignment between cyanobacterial (CalDOX and LepDOX) and plant α-DOXs (OsaDOX and AthDOX) with known

structures. Heme-interacting, catalytic, and substrate-recognition residues are shown as triangles in yellow, red, and blue, respectively.

Table S1. Effect of Triton X-100 (1%, *v/v*) treatment during cell lysis on the Soret peak and oxygen-consumption activities of purified CalDOX and LepDOX.

		Soret peak (nm)	R_z[a]	Relative activity (%)[b]
CalDOX	w/o Triton X-100	414	0.06	100±2.4
	w/ Triton X-100	408	0.10	124±3.3
LepDOX	w/o Triton X-100	414	0.05	100±7.7
	w/ Triton X-100	410	0.08	121±0.6

[a]R_z value, the ratio of absorbance at the Soret peak to the absorbance at 280 nm, represents the heme content normalized by the amount of enzyme (Colas and De Montellano 2004).

[b]Activity was obtained using 2 mM myristic acid based on the oxygen depletion assay.

Table S2. Heme contents of monomers and aggregates of CalDOX and LepDOX.[a]

Enzyme	Oligomeric state	Soret peak (nm)	R_z value[b]
CalDOX	Monomer	404	0.14
	Aggregate	404	0.12
LepDOX	Monomer	406	0.11
	Aggregate	414	0.04

[a]R_z value: the ratio of absorbance at the Soret peak to the absorbance at 280 nm. This represents the heme content normalized by the amount of enzyme (Colas and De Montellano 2004).

[b]Monomeric and aggregated fractions of CalDOX and LepDOX obtained from a size-exclusion chromatographic run were pooled and concentrated and then subjected to the absorbance scan to determine the R_z values.

References

Colas C, De Montellano PR (2004) Horseradish peroxidase mutants that autocatalytically modify their prosthetic heme group: insights into mammalian peroxidase heme-protein covalent bonds. J Biol Chem 279(23):24131-24140. https://doi.org/10.1074/jbc.M401687200
